# The RNA Architecture of the SARS-CoV-2 3′-Untranslated Region

**DOI:** 10.3390/v12121473

**Published:** 2020-12-21

**Authors:** Junxing Zhao, Jianming Qiu, Sadikshya Aryal, Jennifer L. Hackett, Jingxin Wang

**Affiliations:** 1 Department of Medicinal Chemistry, University of Kansas, Lawrence, KS 66047, USA; zhao.junxing@ku.edu (J.Z.); sadixa19@gmail.com (S.A.); 2 Department of Microbiology, Molecular Genetics & Immunology, University of Kansas Medical Center, Kansas, KS 66160, USA; jqiu@kumc.edu; 3 Genome Sequencing Core, University of Kansas, Lawrence, KS 66045, USA; jhackett@ku.edu

**Keywords:** SARS-CoV-2, COVID-19, DMS, DMS-MaPseq, ShapeKnots, DREEM, pseudoknot, three-helix junction, 3′ UTR, minigene

## Abstract

Severe acute respiratory syndrome coronavirus 2 (SARS-CoV-2) is responsible for the current COVID-19 pandemic. The 3′ untranslated region (UTR) of this β-CoV contains essential *cis*-acting RNA elements for the viral genome transcription and replication. These elements include an equilibrium between an extended bulged stem-loop (BSL) and a pseudoknot. The existence of such an equilibrium is supported by reverse genetic studies and phylogenetic covariation analysis and is further proposed as a molecular switch essential for the control of the viral RNA polymerase binding. Here, we report the SARS-CoV-2 3′ UTR structures in cells that transcribe the viral UTRs harbored in a minigene plasmid and isolated infectious virions using a chemical probing technique, namely dimethyl sulfate (DMS)-mutational profiling with sequencing (MaPseq). Interestingly, the putative pseudoknotted conformation was not observed, indicating that its abundance in our systems is low in the absence of the viral nonstructural proteins (nsps). Similarly, our results also suggest that another functional *cis*-acting element, the three-helix junction, cannot stably form. The overall architectures of the viral 3′ UTRs in the infectious virions and the minigene-transfected cells are almost identical.

## 1. Introduction

Since the outbreak of SARS-CoV-2 in December 2019, the virus has infected at least 41 million individuals and caused more than 1.1 million deaths worldwide. SARS-CoV-2 belongs to the β-CoV genus, and is an enveloped ssRNA(+) virus, with a genome length of about 30,000 nucleotides (nts, RefSeq NC_045512.2) [1]. The viral genome bound to the helical nucleocapsid is 5′ capped and 3′ polyadenylated. There are 12 open reading frames (ORFs) from the 5′ to 3′ in the viral genome, namely ORF1a, ORF1ab, spike glycoprotein (S), ORF3a, envelope small membrane protein (E), membrane protein (M), ORF6, ORF7a, ORF7b, ORF8, nucleoprotein (N), and ORF10 [2]. The 5′ two-thirds of the genome has two long ORFs, ORF1a, and ORF1ab that are translated into two polyprotein (pp) precursors, pp1a and pp1ab. The pp precursors are cleaved by viral proteases into 16 nonstructural proteins (nsps), some of which have essential viral functions [2]. For example, nsp12, an RNA-dependent RNA polymerase (RdRp), in pp1ab is required for viral transcription and replication. Nsp12, some other nsps in pp1ab, and certain host factors collectively form the replication transcription complex (RTC), which reads the viral genome template in the 3′ to 5′ direction [3,4]. The viral 3′ UTR contains the first binding site of the RTC as well as multiple *cis*-acting regulatory elements that are essential for the viral genome transcription and replication [5].

Here, we focused on elucidating the structure of the 3′ UTR in the SARS-CoV-2 RNA genome. The sequence from the 3′ of the N gene to the 5′ of the poly(A) tail (nts 29,534–29,870 in RefSeq NC_045512.2, or nts 1–337 in all figures in this article) is defined to be the viral 3′ UTR in this study. This defined 3′ UTR is in accordance with the literature for other β-CoV studies [5], although it contains an open reading frame, ORF10, encoding a 4.4 kDa peptide, of which the function is not completely understood [6]. In a model β-CoV that is permissive to mice, mouse hepatitis virus (MHV), the *cis*-acting RNA elements at the 3′ UTR were reported to be essential for viral transcription and replication [7,8]. Specifically, a bulged stem-loop (BSL) and a pseudoknot in equilibrium were previously proposed to form a molecular switch for the transcription of the 3′ nested set of subgenomic RNAs (sgRNAs) [5]. It was proposed that the pseudoknot formation is required for the binding of the β-CoV canonical RdRp in MHV [8].

**Figure A1:**
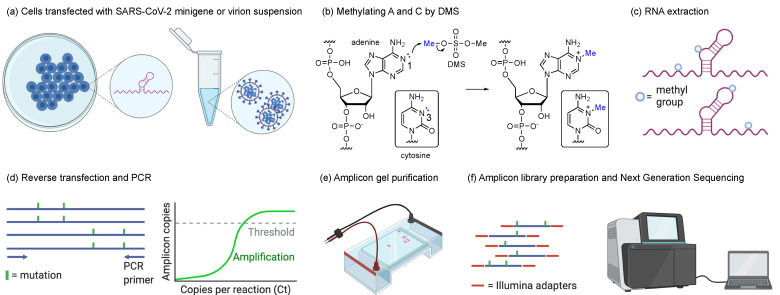
The workflow of DMS-MaPseq in this study. (**a**) BSR T5/7 cells were transfected with SARS-CoV-2 minigene for in vivo DMS-MaPseq. Infectious virions were collected for in-virion DMS-MaPseq. (**b**) Adenines and cytosines in the unpaired region of the transcriptome have a higher probability to be methylated by DMS. (**c**) The DMS-modified RNA was extracted. (**d**) The A- or C-methylated RNA can induce single-point mutations at the methylated nucleobase during the reverse transcription. Amplicons of 895 and 1291 base pairs were amplified by PCR for in-virion and in vivo minigene DMS-MaPseqs, respectively. (**e**) The band was purified by agarose gel electrophoresis. (**f**) The amplicons were ligated with Illumina sequencing adapters and sequenced in at a paired-end mode (150 bp × 2). (**a**,**e**,**f**) are generated by BioRender.com.

To date, efforts have been made to elucidate the SARS-CoV-2 RNA element structures *in silico* [9,10] and by chemical or enzymatic probing for the full or partial RNA genome in virus-infected cells [11,12,13,14] and in vitro [12,15,16,17]. We herein report the RNA structure of SARS-CoV-2 3′ UTR by using a chemical probing strategy, namely dimethyl sulfate (DMS)-mutational profiling with sequencing (MaPseq) [18] in virions and minigene-transfected cells. Although DMS-MaPseq has previously been used to elucidate the SARS-CoV-2 RNA structures in the virus-infected cells, the 3′ UTR was, however, not discussed in that study [11]. DMS is a reactive chemical to RNA nucleobases, resulting in methylations on adenine (A) and cytosine (C). The chemical mechanism of DMS reaction with RNA is different from another commonly used chemical probing strategy, namely selective 2′-hydroxyl acylation analyzed by primer extension (SHAPE), which uses activated esters or amides to acylate the 2′-OH group on the ribose regardless of the nucleobase identity [19]. Both DMS and SHAPE reactions occur at a higher probability to the unpaired nucleotides compared to the paired ones due to the accessibility of the nucleophilic attack. The DMS modification on RNA can be subsequently captured by TGIRT-III reverse transcriptase that induces a single-point mutation at the methylated site [18]. The mutation rate at single-nucleotide resolution can be accurately measured by deep sequencing of the purified amplicon. The normalized DMS activity on each nucleotide is then analyzed and used to match the base pairing and predict possible RNA secondary structures [18]. Because DMS-MaPseq can only recognize the methylation of N1 on purines and N3 on pyrimidines, mutational profiling (MaP) can only be performed for As and Cs but not Gs and Us (see Figure A1 for the DMS-MaPseq workflow).

## 2. Materials and Methods

### 2.1. Cell Culture

BSR-T5/7 cells were a gift from Dr. Peter G. Schultz’s lab at the Scripps Research. Vero E6 cells (CCL-81) were purchased from ATCC. The cells were cultured in Dulbecco’s Modified Eagle Medium (DMEM) containing 10% fetal bovine serum (FBS) and 1× Antibiotic-Antimycotic (Thermo, Waltham, MA, USA) 15240062). BSR-T5/7 cells were also supplemented with 1.0 mg/mL G418 in the medium. The cells were incubated in a humidified cell-culture cabinet at 37 °C and sub-cultured using TrypLe (Thermo 12605036) at 90% confluency at a 1:6 ratio. The cells were passaged at least twice before transfection.

### 2.2. Transfection and DMS Treatment

The minigene plasmid, pUC57-COVID-MG, was constructed by the pUC57 vector and a synthetic gBlock DNA (Integrated DNA Technologies, Coralville, IA, USA) using In-Fusion HD cloning (Takara, Mountain View, CA, USA, 638909). See Supplementary Materials for the full inserted sequence in the pUC57 vector. For minigene transfection, 8 µg pUC57-COVID-MG and 20 µL lipofectamine 2000 (Thermo 11668019) were used for one 10 cm dish of BSR-T5/7 cells according to the manufacturer’s protocol. At 12 h post-transfection, the medium was replenished, and the cells were incubated for 48 h before the DMS treatment. The cells were lifted by TrypLe and the cell pellets were suspended in DMEM in 2% FBS at 6.7 × 10^6^/mL. 2 µL pure DMS (Sigma-Aldrich, St. Louis, MO, USA, D186309) was added into pre-warmed 200 µL cell suspension and mixed by repetitive pipetting. After incubation for 5 min, the DMS reaction was quenched by 100 µL 50% β-mercaptoethanol in PBS. The cells were immediately pelleted and the DMS-modified total RNA was extracted by Qiagen (Hilden, Germany) RNeasy Mini kit according to the manufacturer’s protocol with DNase I treatment. In the DMS-untreated sample, the untreated-RNA was prepared following the same protocol in parallel, except that DMS was not added.

### 2.3. SARS-CoV-2 Propagation and DMS Treatment

We obtained icSARS-CoV-2-mNG, an infectious recombinant SARS-CoV-2 that expresses a bright monomeric yellow-green fluorescent protein (mNeonGreen) by incorporating a codon-optimized reporter gene into the ORF7a of the viral genome [20], from Drs. Shi and Menachery through the University of Texas Medical Branch’s World Reference Center for Emerging Viruses and Arboviruses. The virus was passaged in the Vero E6 cells once and titrated using a plaque assay in the Vero E6 cells [21]. A biosafety protocol to work on SARS-CoV-2 in the biosafety level 3 laboratory was approved by the Institutional Biosafety Committee (IBC) of the University of Kansas Medical Center. The titer of the icSARS-CoV-2-mNG stock was 3 × 10^7^ plaque-forming units (pfu)/mL. 2 µL DMS was added into a pre-warmed 200 µL virus suspension and incubated for 4 min at 37 °C before quenching with 100 µL 50% β-mercaptoethanol in PBS followed by addition of 3 mL TRIzol (Thermo 15596026). RNA extraction was performed according to the manufacturer’s protocol. Similarly, a DMS-untreated sample was prepared in parallel without the addition of DMS.

### 2.4. Amplicon Library Preparation and Next-Generation Sequencing

All samples were prepared in duplicates. DMS-treated and -untreated RNA samples were reversely transcribed according to the literature [22] except at 55 °C. In both in vivo minigene and in-virion DMS samples, the reverse transcription primer 5′-TTTTTGTCATTCTCCTAAG-3′ was used. An amplicon size of ~1300 bp was chosen using the primer pairs: pUC_fw 5′-ATTAAAGGTTTATACCTTCCCAGG-3′, pUC_rv TTTTTGTCATTCTCCTAAGAAGCT; virion_fw 5′-GAGCAAAATGTCTGGTAAAGGC-3′, virion_rv 5′-TAAGAAGCTATTAAAATCACATGGGG-3′. The cycle number for the PCR was chosen as the end of the exponential stage judged by electrophoresis to avoid over-amplification (in vivo minigene DMS ~34 cycles, in-virion ~21 cycles). The amplicons were purified by agarose gel electrophoresis. For the full sequence of the pUC57-COVID-MG, see Supplementary Materials. The DNA amplicon samples were checked with the High Sensitivity (HS) dsDNA Qubit assay (Thermo) for quantification and Agilent (Santa Clara, CA, USA) TapeStation gel analysis for the amplicon quality and determination of the amplicon base-pair size. The sequencing libraries were constructed using 100–500 ng of amplicon DNA using the Illumina (San Diego, CA, USA) DNA Prep sequencing library kit. The sequencing library construction includes tagmentation of the amplicon DNA using a bead-based transposome complex to simultaneously fragment and tag the DNA with adapter sequences. Following tagmentation, unique index adapters are added in a PCR amplification step to the ends of the DNA fragments. The constructed sequencing libraries were quantified and validated with Qubit and TapeStation assays. After pooling the sequencing library preps together equally by ng amount, the nM concentration of the pool was verified with an Illumina Library Quant qPCR assay (Roche (Basel, Switzerland)). An Illumina NextSeq 550 system was used to generate paired-end, 150-base sequence reads from the multiplexed libraries at a depth of 15 × 10^6^ reads per amplicon library. Base-calling was carried out by the instrument’s Real-Time Analysis (RTA) software. The base call (bcl) files were demultiplexed and converted to compressed FASTQ files by bcl2fastq2. The FASTQ files for the DMS-MaPseq in this study can be downloaded from NCBI Sequence Read Archive (SRA) under BioProject accession # PRJNA669862.

### 2.5. DMS-MaPseq and DREEM Analysis Pipelines

The quality of the FASTQ files was checked with FastQC [23] before and after trimming the Illumina adapter sequences from the reads (Cutadapt, integrated with TrimGalore [24]). The trimmed paired-end reads were then mapped to the template sequence to calculate the mutation rate using an integrated software package, ShapeMapper2 [25]. The mutation rate of G, U, and primer binding sites was changed into “unavailable”, or −999, before being analyzed by SuperFold v1.0 [26]. SuperFold predicts stable structured regions based on a 55-bp window median of the DMS reactivity (<0.3) and Shannon entropy (<0.04) [27]. The python program Superfold.py in the SuperFold v1.0 package was modified (available in Supplementary Materials) to integrate a free energy minimization function by using RNAstructure’s ShapeKnots program [28]. The in vitro DMS-MaPseq data set was downloaded from Gene Expression Omnibus (GEO) database, under the accession GSE151327 [12]. The DREEM analysis is performed in a 70–200 nucleotide range according to literature using the un-trimmed FASTQ files [29]. Adapter-trimming and mapping using bowtie2 are integrated into the DREEM program [29]. The DREEM program filters the qualified reads and converts them into bit vectors based on the mutation rate (if mutation rate > 0.5%, converts to 1; otherwise, 0), and categorize them into 2–4 clusters. DREEM predicts stable structures in a similar approach to SuperFold, except uses 5% of the nucleotides in the region of interest for normalizing DMS reactivities. To integrate the RNA folding program, ShapeKnots, the python program EM_ExpandFold.py in the DREEM package was modified (available in Supplementary Materials).

## 3. Results

### 3.1. DMS-MaPseq Uncovered almost Identical SARS-CoV-2 3′ UTR RNA Structures in Minigene-Transfected Cells and Infectious Virions

**Figure 1:**
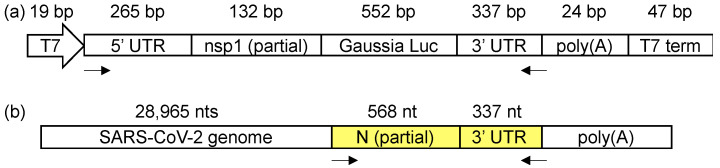
(**a**) The SARS-CoV-2 minigene construct containing the 5′ and 3′ untranslated regions (UTRs) and a short poly(A) tail. (**b**) The amplicon location (highlighted in yellow) in the SARS-CoV-2 genome for the in-virion DMS-MaPseq. The arrows indicate the primer-binding sites for the amplicons.

We used two systems for the viral 3′ UTR structural probing: (1) SARS-CoV-2 UTR minigene-transfected cells, and (2) infectious virions. We first constructed a SARS-CoV-2 UTR minigene plasmid (Figure 1a) using a replication vector, pUC57. The target SARS-CoV-2 UTR minigene reporter sequence was inserted in a T7 transcription cassette. The whole T7 cassette contains, from the 5′ to 3′, the T7 promoter, the 5′ of SARS-CoV-2 genome (1–397 bp), *Gaussia* luciferase, the SARS-CoV-2 3′-UTR (nts 29,534–29,870), a 24-nt long poly(A) sequence, and the T7 terminator sequence. 1–397 nt of SARS-CoV-2 contains both the 5′ UTR (nts 1–265) and part of the first nsp1 gene in pp1a (nts 266–397). The pUC57 plasmid does not possess any mammalian cell expression element, and, therefore, the transcription of the genes is solely controlled by the T7 promoter. The plasmid was transfected into a mammalian T7-expressing cell line, BSK-T5/7, which is commonly used for RNA viral replication studies [30]. At 48 h after the transfection, the DNA template in the T7-transcription cassette was transcribed in the cytosol, generating an RNA sequence that contains the viral 3′ UTR. Since no other viral genes were co-transfected into the cells, this minigene RNA is not likely to possess a 5′-cap and only contains a short, 24 bp, poly(A) tail, which makes the RNA unable to undergo replication or gene expression. For chemically probing the structure of this in vivo transcribed viral UTR minigene transcript, the transfected BSR-T5/7 cells were transiently treated with DMS at 48 h after the transfection. The DMS-modified RNA was then converted into the complementary DNA (cDNA) with TGIRT-III reverse transcriptase using a gene-specific reverse transcription primer, which binds to the junction of the 3′ UTR and the poly(A) tail. A 1291 bp amplicon is amplified by PCR, which covers the whole minigene except for the poly(A) tail (Figure 1a). The gel-purified amplicon from both DMS-treated and untreated samples, in duplicates, was then prepared as a DNA amplicon library for deep sequencing (>15 million reads per amplicon). The DMS-induced mutations were mapped and analyzed by a well-established bioinformatics pipeline, ShapeMapper2 [25]. ShapeMapper2 was originally designed for the SHAPE-MaP experiment but is also adaptable for DMS-MaPseq analysis as in our experiment [25].

In parallel, the SARS-CoV-2 3′ UTR in the infectious virions was also chemically probed by DMS. The capsid proteins S, E, M, and N are not known to specifically interact with the viral RNA in the 3′ UTR [2]; the known 3′ UTR-interacting viral proteins, such as nsp8 and nsp12 [8], or the host proteins are not present in the isolated infectious virions. Therefore, the in-virion DMS-MaPseq will uncover the RNA secondary structures without the interference of the host proteins or the viral nsps. The infectious virus was produced by transfecting the cDNA clone of SARS-CoV-2 into the Vero E6 cells [20]. The virus generated in this approach maintained the infectivity at ~3 × 10^7^ pfu/mL. The viral suspension in the medium was treated with DMS, followed by the same protocol as the in vivo minigene DMS-MaPseq described above, except that the forward primer binds within the N gene (Figure 1b).

**Figure 2:**
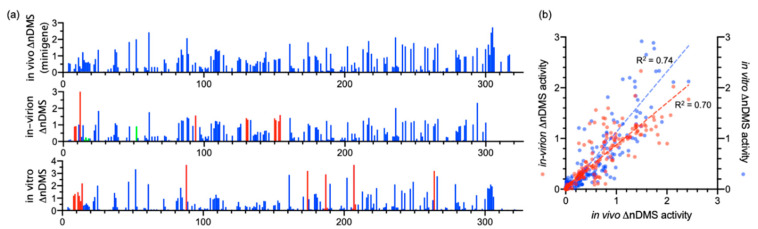
(**a**) The in vivo minigene, in-virion, and in vitro normalized dimethyl sulfate (DMS) activity (ΔnDMS) profiles of the SARS-CoV-2 3′ UTR. Compared to the in vivo minigene, the significantly higher or lower ΔnDMS activities are labeled in red and green, respectively (>95% confidence in *Z*-factor test and >1.5-fold ΔnDMS activity change, see Table S1 for details). The activity data are not available for the primer-binding regions, i.e., nts 319–327, 302–327, and 311–327 in the in vivo minigene, in-virion, and in vitro DMS-MaPseqs, respectively. (**b**) The correlations between the in vivo minigene and in-virion or in vitro ΔnDMS activities. The out-of-range data points are omitted in the figure. The in vitro DMS-MaPseq was re-analyzed using the data from Ref. [12] with the ShapeMapper2 pipeline (see Methods).

**Figure 3:**
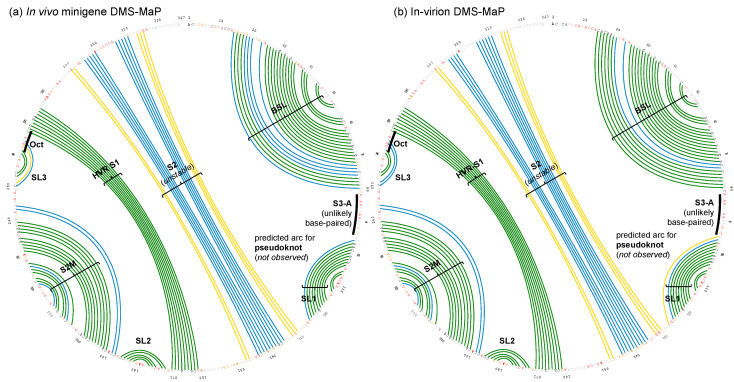
Arc plots of the most possible base-pairing pattern for SARS-CoV-2 3′ UTR. A colored arc represents a base pair with the following probability: 100% > green ≥ 80% > blue ≥ 30% > yellow ≥ 10%. By definition, ΔnDMS is high when ΔnDMS > 0.85, medium 0.40 < ΔnDMS ≤ 0.85, or low ΔnDMS ≤ 0.40, and the corresponding nucleotide symbol is colored red, orange, or black, respectively. The nucleotides of G and U, or within PCR primer-binding regions, where the DMS data is not available, are colored grey. The arc plots were generated by the SuperFold software package [26] from the experiments (**a**) in vivo minigene DMS-MaPseq, and (**b**) in-virion DMS-MaPseq. See Figure S2 for full arc plots with all possible base-pairing possibilities.

**Figure 4:**
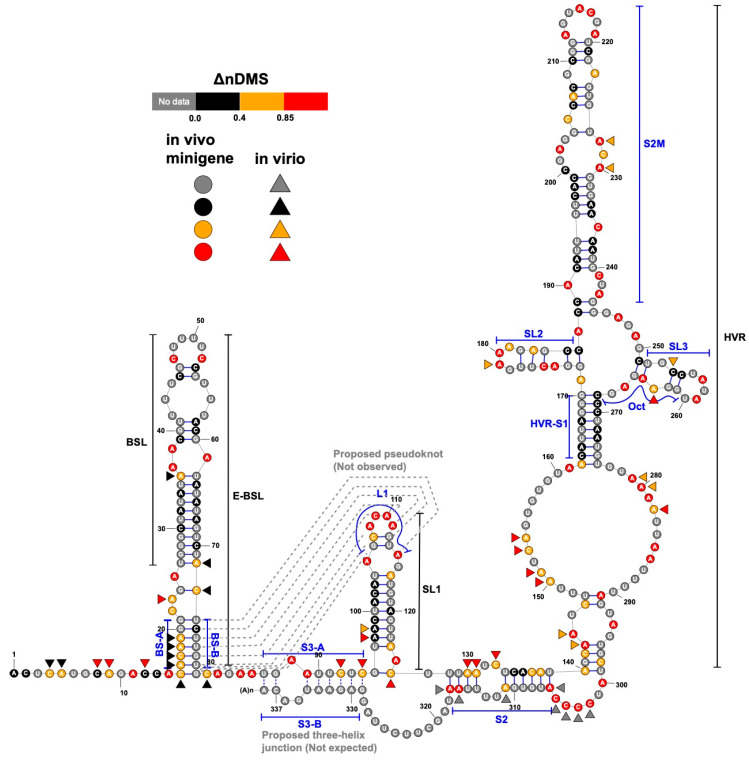
A consensus structure derived from the in-virion and in vivo minigene DMS-MaPseqs. The ΔnDMS reactivity for each nucleotide from the in vivo minigene DMS-MaPseq is colored according to the legend on the RNA backbone, with the in-virion DMS activity drawn aside if it belongs to a different reactivity group. The occurrences of the base pairing for BS-B–L1 and S3-A–S3-B (dotted lines) are not supported in this study.

We compared our in vivo minigene and in-virion DMS-MaPseq data with the in vitro data in the literature [12]. We used ShapeMapper2 pipeline to re-analyze the published in vitro DMS-MaPseq data. The normalized ΔDMS (normalized [DMS(+) − DMS(−)] in mutation rate, or ΔnDMS [31]) in single-nucleotide resolution between in vivo minigene and in-virion or in vitro DMS-MaPseqs are mostly consistent by *Z*-factor test (deltaSHAPE [32], Figure 2a). The nts 8–12 in the viral 3′ UTR in minigene-transfected cells demonstrated a significantly lower ΔnDMS signal than that in virions and in vitro, likely indicating a potential host protein-binding site (Table S1) [32]. There is also a reasonably high-degree correlation of ΔnDMS activities between in vivo minigene-in-virion or in vivo minigene-in vitro DMS-MaP in linear regression (R^2^ > 0.70, Figure 2b). We used a software package, SuperFold [26], which integrates the base-pairing and RNA secondary structure prediction (ShapeKnots [28]) algorithms (see Methods). The arc plots, representing the base-pairing probabilities, generated in both in vivo minigene and in-virion settings are highly similar (Figure 3), resulting in almost identical structures (Figure 4). This 3′ UTR structure is overall the same as the structure derived from SHAPE-MaP in literature [13]. The stem regions of BSL, stem-loop 1 (SL1), the stem-loop II-like motif (S2M), and the hyper-variable region (HVR)-S1 all have low DMS reactivities, consistent with the in vivo SHAPE-MaP data [12,13]. Some subtle differences were found between DMS- and SHAPE-MaPs. For example, in the HVR, two additional small stem-loops (SL2 and SL3) are predicted in DMS-MaPseq but not SHAPE-MaP [12,13]. However, these structures in the HVR are unstable, as discussed in the following section. The SARS-CoV-2 minigene also contains the viral 5′ UTR. The 5′ UTR structure predicted from the pipeline is identical to the reported one derived from in vivo DMS-MaPseq in the virus-infected cells [11] (Figure S1).

### 3.2. DREEM Analysis Uncovered an Unexpected Lack of the Pseudoknot and Three-Helix Junction in the 3′ UTR

**Figure 5:**
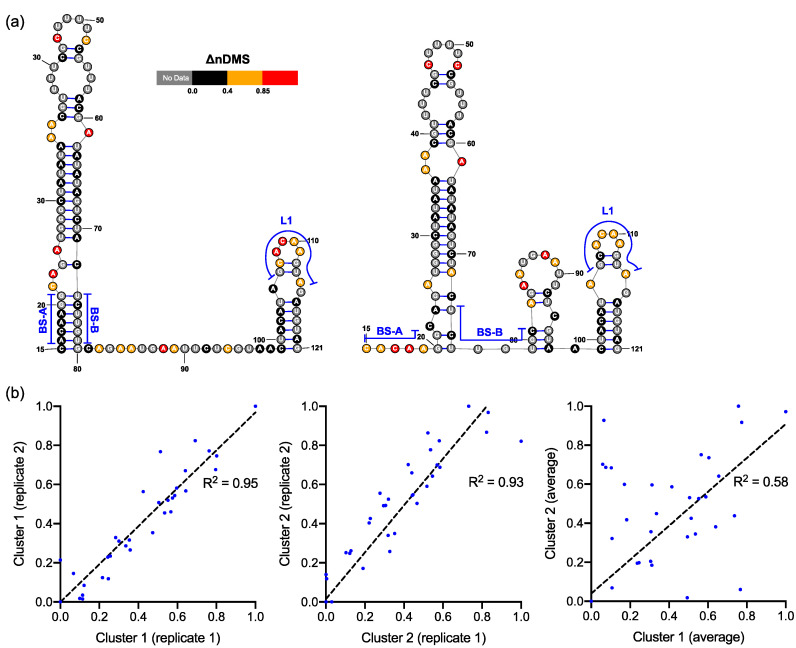
(**a**) Two predominant conformations of bulged stem-loop (BSL) and stem-loop 1 (SL1) uncovered by the DREEM analysis on the DMS-MaPseq reads that cover >95% of the region. The illustrated structures were derived from replicate 2 data set. (**b**) Correlation in ΔnDMS activity profiles for the same structural cluster between the replicates and between two clusters.

In both the in vivo minigene and in-virion DMS-MaPseq, the pseudoknot with base pairs of L1–BS-B was not predicted as a possible conformer (Figure 3 and Figure 4 and Figure S2), although they are complementary Watson-Crick pairs. The base pairing of BS-A–BS-B and BS-B–L1 cannot simultaneously occur. Therefore, the extended BSL (E-BSL) with BS-A–BS-B and a pseudoknot with BS-B–L1 are mutually exclusive, which is the basis of the molecular switch hypothesis [6]. In theory, in case the pseudoknot forms, the DMS activities for L1 and BS-B should both be low in an RNA helix, leaving the high DMS reactivities on the unpaired BS-A segment. On the contrary, in-virion DMS-MaPseq clearly showed low DMS activities on BS-A (Figure 3), thereby exclude the possibility of the pseudoknot to form as a major conformer. The in vivo minigene DMS-MaPseq, on the other hand, showed medium-to-high DMS activities on both segments BS-A and L1 (Figure 3 and Figure 4). This finding led us to ask: is it possible that the 3′ UTR is in an equilibrium between a more favorable E-BSL and a less favorable pseudoknotted conformation in the minigene-transfected cells, and the data for the predominant E-BSL masks the prediction of a pseudoknot using the conventional DMS-MaPseq analysis? To answer this question, we applied a recently developed algorithm, DREEM, to isolate less populated but stable RNA conformers [29]. Compared to other programs that can uncover different structural groups in a mixture of RNA conformations [33,34], DREEM is advantageous because it clusters the structural groups in the first step and analyzes the RNA structure within the individual groups [29]. The DREEM algorithm groups multiple DMS-modified sequencing reads that cover 95% of the region of interest into a certain number (2–4) of clusters. RNA conformation of each structural cluster is subsequently predicted using a free energy minimization program, ShapeKnots [28], in the RNAstructure software package [31] (Figure 5, see Methods). If a pseudoknot and an E-BSL both stably exist, DMS-modification patterns for both conformers should be identified by DREEM. Unexpectedly, we did not observe a structural cluster with a DMS-modification pattern that matches the pseudoknotted conformation. Instead, the DREEM program consistently grouped the sequencing reads into two structural clusters in ~1:1 ratio as shown in Figure 5a. In both clusters, L1 maintains medium-to-high DMS activity (Figure 5a). Structural cluster 1 is identical to the structure derived from conventional DMS-MaPseq analysis (Figure 4). In structural cluster 2, the DMS activities are high in BS-A likely because a new stem-loop forms, which partially involves BS-B, making BS-A overhung and unpaired (Figure 5a). The DMS activities are consistent between the two replicates within each cluster (R^2^ > 0.93) and are less correlated between the two clusters (R^2^ = 0.58) (Figure 5b).

Besides BSL and SL1, a complex HVR and a three-helix junction that contains SL1, S2, and S3 are both predicted in SARS-CoV-2 [10] (Figure 4). The three-helix junction is the last RNA element before the poly(A) tail. Because the S3-B segment locates at the very end of the 3′ and overlaps with the primer-binding region for the amplicon, no valid DMS data were collected for S3-B. However, in both the in-virion and in vivo minigene DMS-MaPseqs, the DMS activities in S3-A are both rated from medium to high (Figure 3 and Figure 4), suggesting that the base pairing between S3-A and S3-B is unlikely. This conclusion is also supported by a recent RNA-RNA interactome study in SARS-CoV-2 in a psoralen crosslinking approach [35]. In this study, a long-range interaction between S3-B and 5′ UTR is captured, suggesting that S3-A is unpaired and the three-helix junction is probably not formed [35]. For the HVR, because the region is highly dynamic, we cannot obtain a consistent DREEM result except for the S2M and S1 segments (Figures S3 and S4). The structure shown in Figure 4, therefore, merely reflects the averaged conformation and is not accurate in the HVR in the minigene-transfected cells. It is also worth noting that the in vivo minigene RNA, a T7-transcribed artificial transcript, uncapped and unfunctional, may not represent the structure of a functional viral transcript in the infected cells.

## 4. Discussion

**Figure 6:**
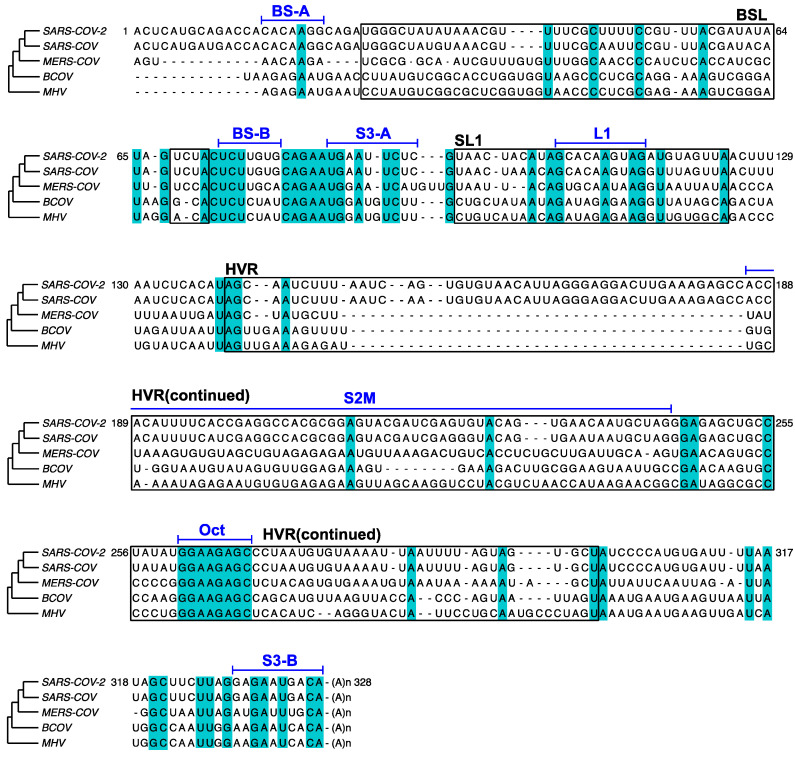
Comparison of the 3′ UTR sequences among SARS-CoV-2 (RefSeq NC_045512.2), SARS-CoV (RefSeq NC_004718), MERS-CoV (NC_019843), BCoV (U00735), and MHV (NC_048217). Major RNA elements, BSL, SL1, and HVR, are boxed and annotated in black. Shorter RNA segments are annotated in blue above a bar aligned with the sequences. Conserved nucleotides are shaded in cyan. 717 nucleotides at the 5′ of MERS 3′ UTR are omitted for clarity.

In this study, the in vivo minigene and in-virion DMS-MaPseq derived RNA structures for SARS-CoV-2 3′ UTR were not predicted to possess a pseudoknot. Our finding is consistent with the previous thermodynamic study on the MHV 3′ UTR, in which the pseudoknotted conformation was only observed with limited stability at 25 °C [36]. The MHV pseudoknot is fully melted and transformed into the E-BSL conformer in vitro at 37 °C [36]. All the DMS-MaPseq experiments for SARS-CoV-2 3′ UTR in this study were also conducted at 37 °C. It was suggested in the literature that the formation of the pseudoknot depends on the ionic conditions and the presence of cellular proteins [37]. This result also agrees with the prediction from the recent SHAPE-MaP [12,13,14,15], psoralen crosslinking [35], and NMR spectroscopy data [17]. On the other hand, bioinformatic analyses argue the existence and the essential function of the pseudoknot [7]. In the comparison of the RNA sequences in BSL and SL1 segments among β-CoV species (Figure 6), BS-B–L1 base pairing is phylogenetically conserved. The existence of the equilibrium between a pseudoknot and BSL are supported by quantitative covariation analysis using RNA Structural Covariation above Phylogenetic Expectation (R-scape) algorithm (RF11065, Rfam database) [38]. In MHV, the function of the pseudoknot structure is supported by reverse genetic studies. The pseudoknot was challenged by mutagenesis for the viral replicability [7]. Several mutations that destabilize BS-A–BS-B or BS-B–L1 make the mutant MHV strain non-replicating; whereas a mutation on all three RNA segments, which possibly retains the equilibrium between the E-BSL and pseudoknot partially rescued the viral replicability [7]. It is also worth noting that in the amplicon library preparation step for both in vivo and in-virion DMS-MaPseqs, the chemical probing information on the RNA was first passed on to cDNAs, and subsequently amplified by PCR. It is known that PCR amplification can possibly bring bias and eliminate minor species [39]. Together, our results probably suggested that the pseudoknot forms in a very low abundance in the infectious virions or the minigene-transfected cells. In other words, given the compelling supportive evidence from reverse genetics and phylogenetic covariation analysis, the lack of pseudoknot in our observation is probably a reflection of an unfavorable experimental condition for the pseudoknot formation. A possible hypothesis is that the viral proteins might play a role in inducing the pseudoknotted conformation, i.e., the pseudoknot only forms transiently when RTC binds to the 3′ UTR in an “induce-fit” model. The RTC was, however, not included in our minigene or in-virion systems. In the future, detailed DREEM analysis on DMS- or SHAPE-MaP data from the virus-infected cells containing functional RTCs will be performed.

In MHV, the HVR is nonessential for viral RNA synthesis. HVR can be deleted from the viral 3′ UTR without affecting viral replication in the cell culture, albeit the HVR-deletion MHV strain has lower pathogenicity in mice [40]. Some subregion of the HVR is conserved among β-CoV, such as the stable S2M [10] (Figure 6). From the crystal structure analysis of SARS-CoV S2M, it is proposed that this RNA element may bind to the host’s eukaryotic translation initiation factor 1A (eIF-1A) and facilitate hijacking the host protein synthesis machinery [41]. The HVR also contains a conserved octanucleotide (Oct) sequence with an unknown function [10] (Figure 6). S3 helix was shown to be essential for the MHV viability [40,42] and phylogenetically conserved [8] (Figure 6). However, our results argue that the formation of S3 is structurally unstable. In addition, we analyzed 3′ UTR sequences from all 11,704 SARS-CoV-2 sequence records that contain the full 3′ UTR and a poly(A) tail collected from December 2019 to 6 November 2020 in Global Initiative on Sharing All Influenza Data (GISAID) database and discovered that 8.3% of the reads have partially or fully truncated S3-B (see Supplementary Materials for the GISAID sequence names; Figure S5 for the whole 3′ UTR mutational profile from the clinically isolated viruses). This result may suggest that the S3 helix is also not genetically stable in the SARS-CoV-2 genome although the base-calling at the genome ends in sequencing data is not fully accurate [38]. Therefore, the function of the S3-helix must be evaluated carefully in reverse genetic studies for SARS-CoV-2. A recent long-range RNA-RNA interactome study demonstrated that S3-B can base-pair with 5′ UTR, which is also present in our minigene system or isolated virions, resulting in a cyclic genome instead of a short-range, three-helix junction structure within the viral 3′ UTR [35].

Our result also demonstrated that the in-virion 3′ UTR RNA structure is generally identical to the structures derived from minigene-transfected cells and virus-infected cells in the literature [13]. This implies that the driving force of the predominant viral RNA secondary structure formation at the 3′ UTR is base pairing, not the interaction between the RNA and host or viral RNA-binding proteins. It is, however, observed that the viral BSL-SL1 segment can adopt more than one conformation in minigene-transfected cells by a detailed DREEM analysis. This indicates that the host proteins play a role in folding the RNA structure into a less favorable conformation in the BSL-SL1 region. In the minigene RNA, the predominant structures of the viral 5′ and 3′ UTRs are highly consistent with the structure derived from in vivo DMS- [11] and SHAPE-MaP [13] in the virus-infected cells. Therefore, we envision that the SARS-CoV-2 UTR minigene system probably preserves the RNA regulatory elements for viral replication, which paves the way in its use to construct a functional SARS-CoV-2 minigenome replicon assay.

## Supplementary Materials

The following are available online at https://www.mdpi.com/1999-4915/12/12/1473/s1, Figure S1: SARS-CoV-2 5′ UTR structure derived from in vivo minigene DMS-MaPseq, Figure S2: Arc plots of all predicted possible base pairs for SARS-CoV-2 3′ UTR, Figure S3: The predominant structures (>73%) of the S2M segment in the HVR determined by the DREEM analysis, Figure S4: DREEM analysis on the whole HVR does not yield a predominant conformer, Figure S5: The mutation rate in each 3′ UTR nucleotide from 11,704 clinically isolated SARS-CoV-2 specimens, Figure S6: The mutation rates, read depths, and reactivity distribution of the in vivo minigene, in-virion and in vitro DMS-MaPseq using the ShapeMapper2 pipeline, Table S1: The normalized DMS activities (ΔnDMS) and standard errors in the 3′ UTR under different DMS settings, The SARS-CoV-2 minigene plasmid (pUC57-COVID-MG) sequence, The terminal command lines for deltaSHAPE and DREEM analysis in BSL/SL1 RNA segments with Parallel [43], Names of the SARS-CoV-2 sequences that are truncated in S3-B (collected and deposited in GISAID database on or before 6 November 2020), modified python programs Superfold.py (for SuperFold) and EM_ExpandFold.py (for DREEM).
