# Supplementary material for: The RNA Architecture of the SARS-CoV-2 3′-Untranslated Region
Source: Viruses. 2020 Dec 21;12(12):1473. doi: 10.3390/v12121473 (PMC7766253; doi:10.3390/v12121473)
Supplement: Supplementary file 1 [file viruses-12-01473-s001.zip › RNA_3UTR_v2_SI.docx]

**Figure S1.** SARS-CoV-2 5’ UTR structure derived from the *in vivo* minigene DMS-MaPseq. Because the forward primer for the amplicon overlaps with the 5’ part of the SL1 (nts 5–18), the structure of SL1 cannot be predicted accurately.

(a)


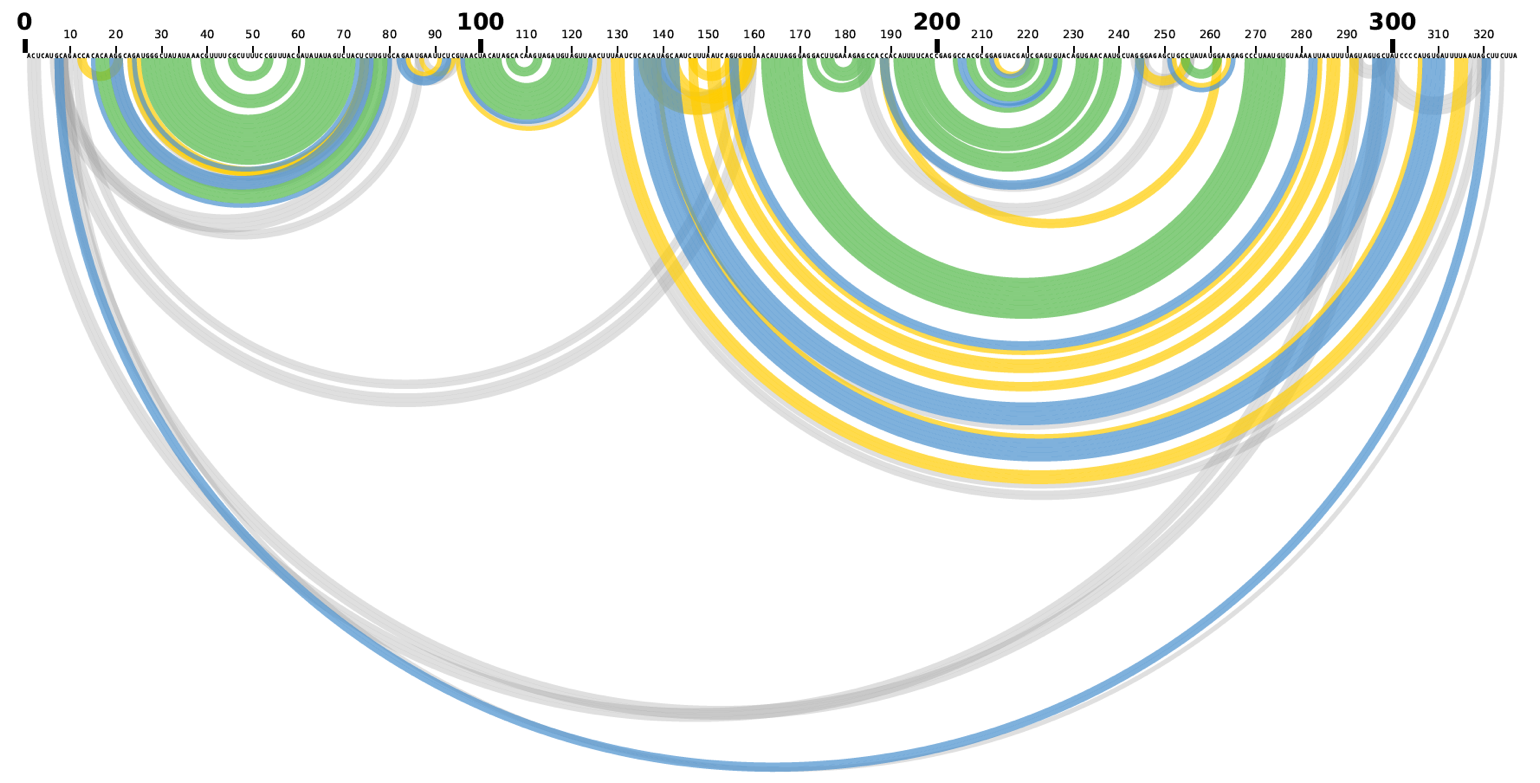


(b)


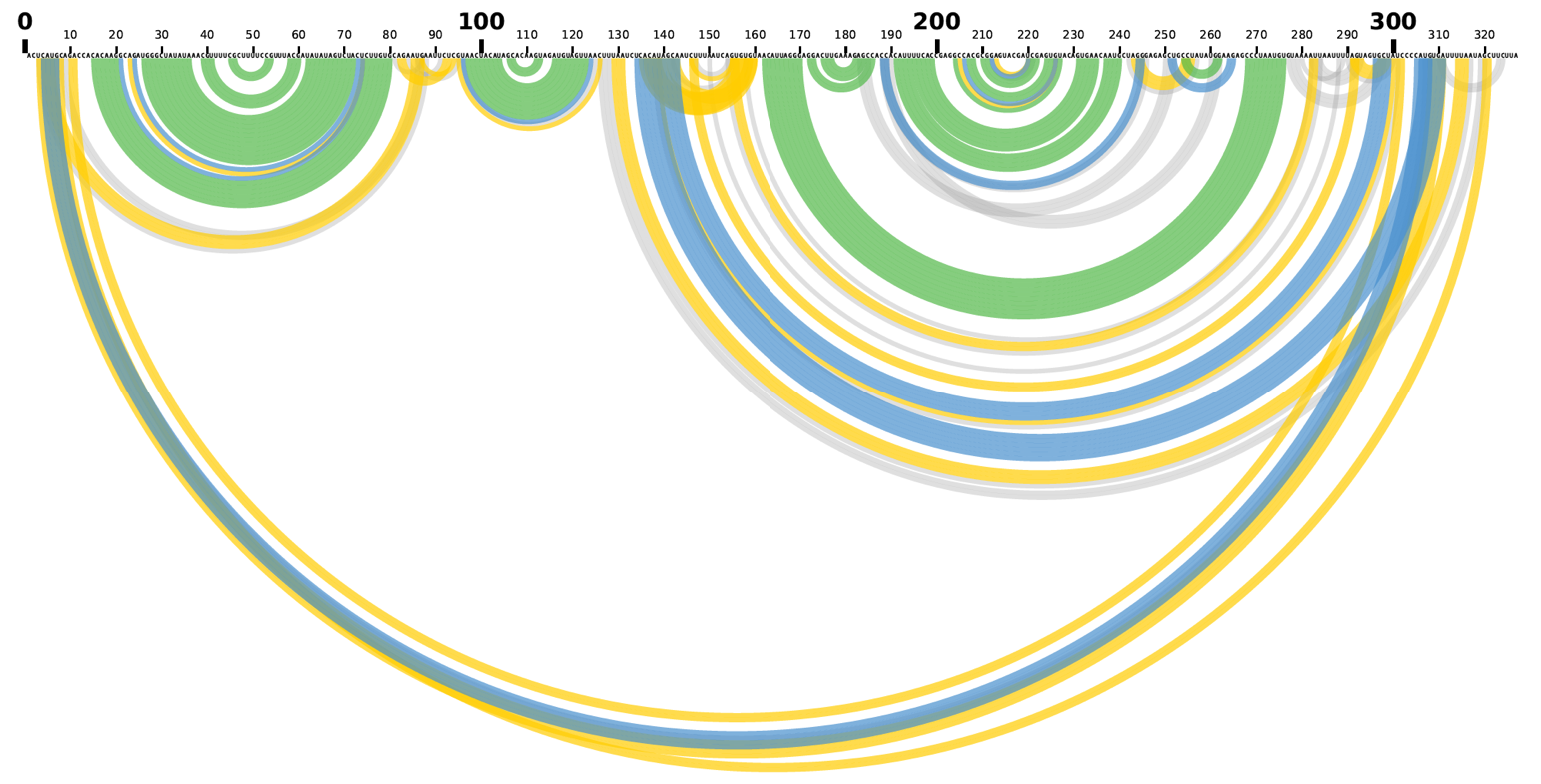


**Figure S2.** Arc plots of all predicted possible base pairs for SARS-CoV-2 3’ UTR. An arc represents a base-pair with the following probability: green > 80%, blue > 30%, yellow > 10%, and gray > 3%. The arc plots were generated by SuperFold software package [1] from the experiments (a) *In vivo* minigene, and (b) in-virion DMS-MaPseq. The nucleotide numbers 1-327 in the figure correlate to SARS-CoV-2 reference genomic sequence, nucleotides 29,534-29,860 (RefSeq NC_045512.2).

**Figure S3.** The predominant structures (> 73 %) of the S2M segment in the HVR determined by the DREEM analysis [2]. The *in vivo* minigene (left) and in-virion (right) S2M structures are identical with the result derived from ShapeMapper2 analysis followed by SuperFold prediction. The numbering of the nucleotides is consistent with Figure S2.

**Figure S4.** DREEM analysis on the whole HVR does not yield a predominant conformer. The figure illustrates two example conformers from the *in vivo* minigene (left) and in-virion (right) DMS-MaPseq. The replicate results are not consistent (not shown) indicating an arbitrary clustering in DREEM probably due to a dynamic equilibration in the HVR. The numbering of the nucleotides is consistent with Figure S2.

**
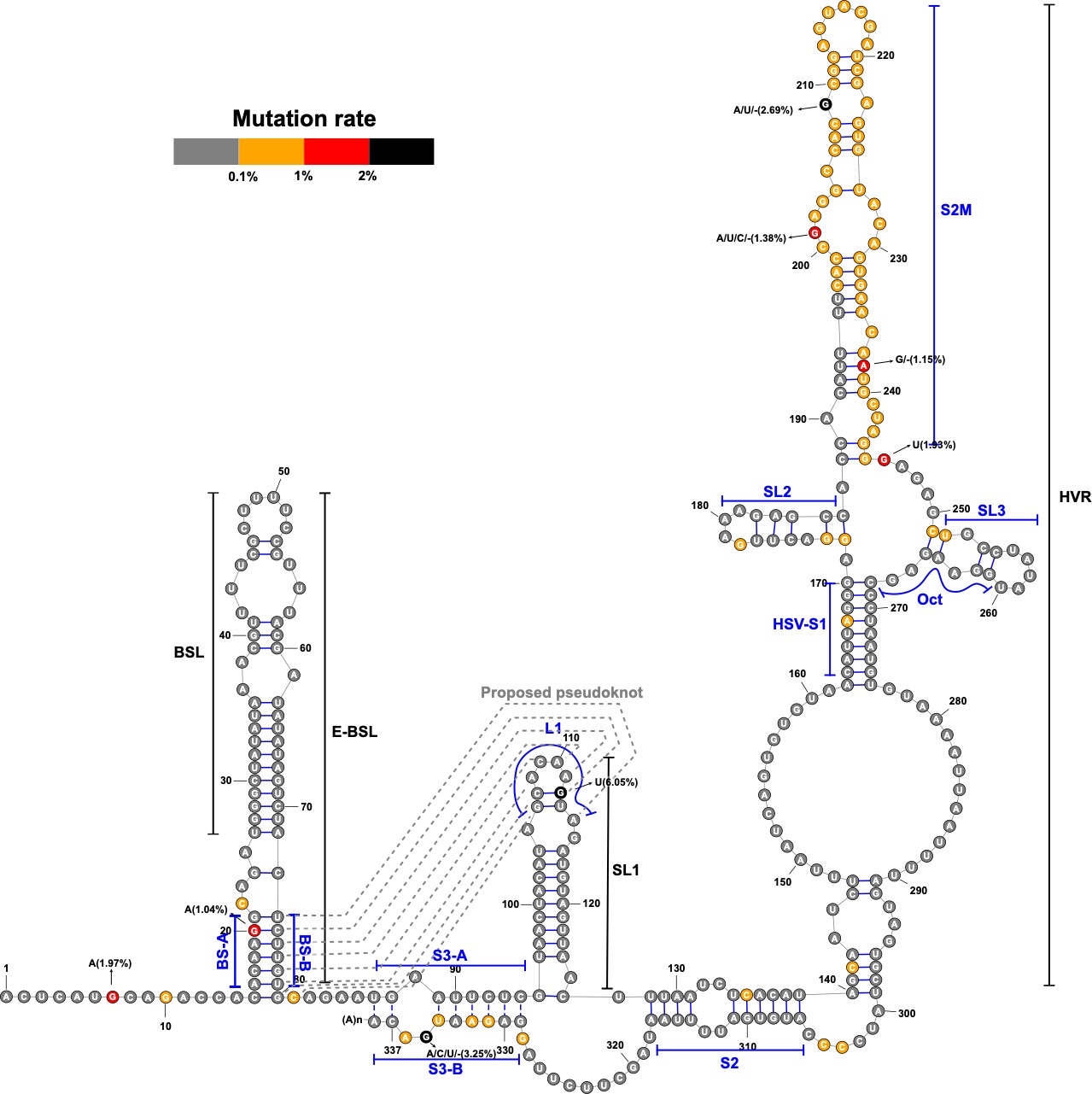
**

**Figure S5.** The mutation rate, compared to the RefSeq NC_004718, in each 3’ UTR nucleotide from 11,704 clinically isolated SARS-CoV-2 specimens. The nt 338 belongs to the poly(A) tail. All records analyzed here must have at least 10-nt long poly(A) tail. The in-virion SARS-CoV-2 3’UTR structure derived from this study is used here with RNA elements or segments annotated in lines that are close to the indicating nucleotides.

(a) *in vivo* minigene DMS-MaPseq


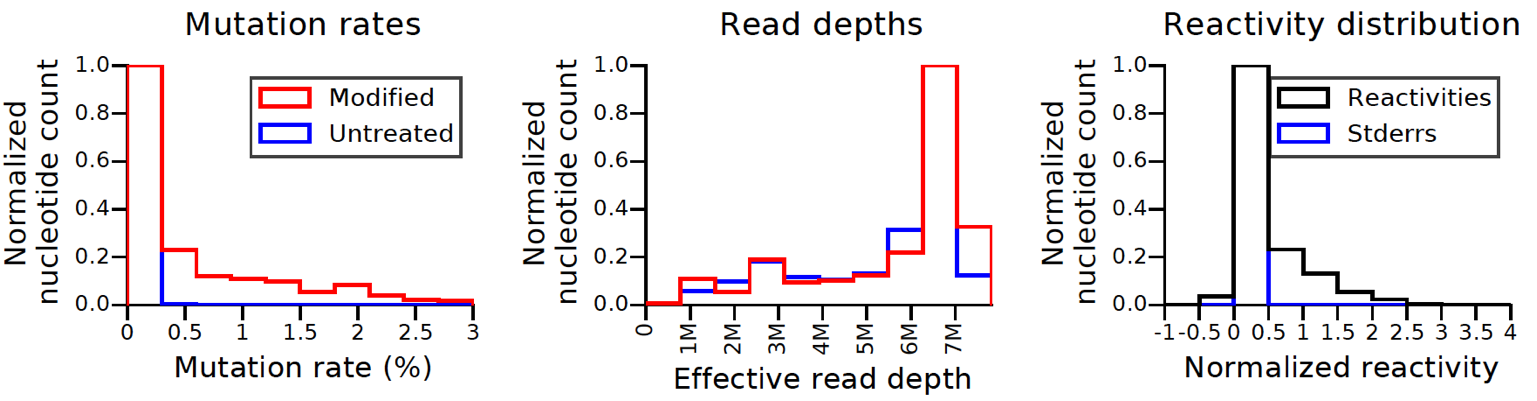


(b) in-virion DMS-MaP seq


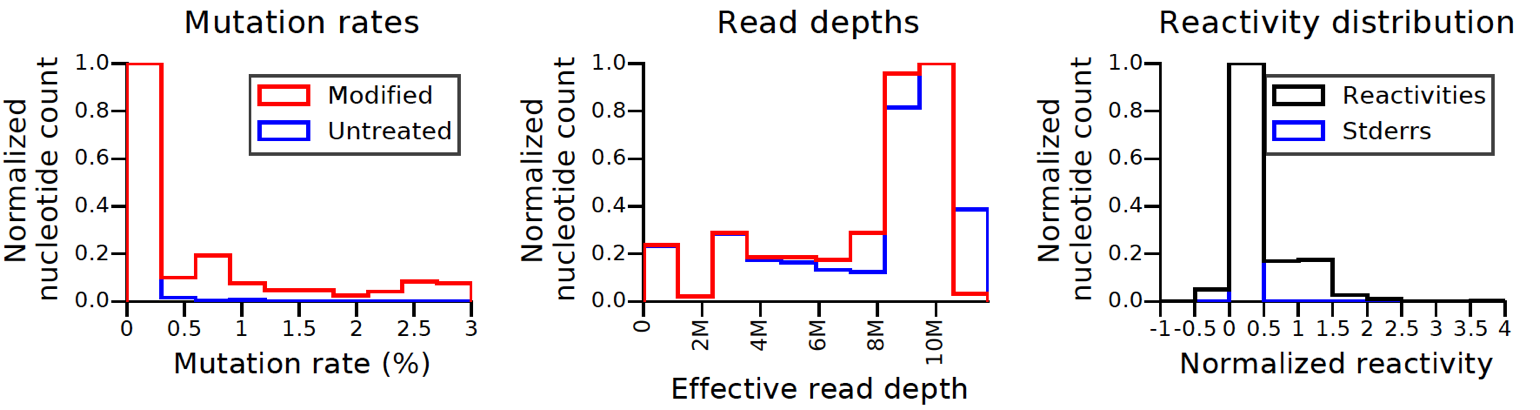


(c) *in vitro* DMS-MaPseq


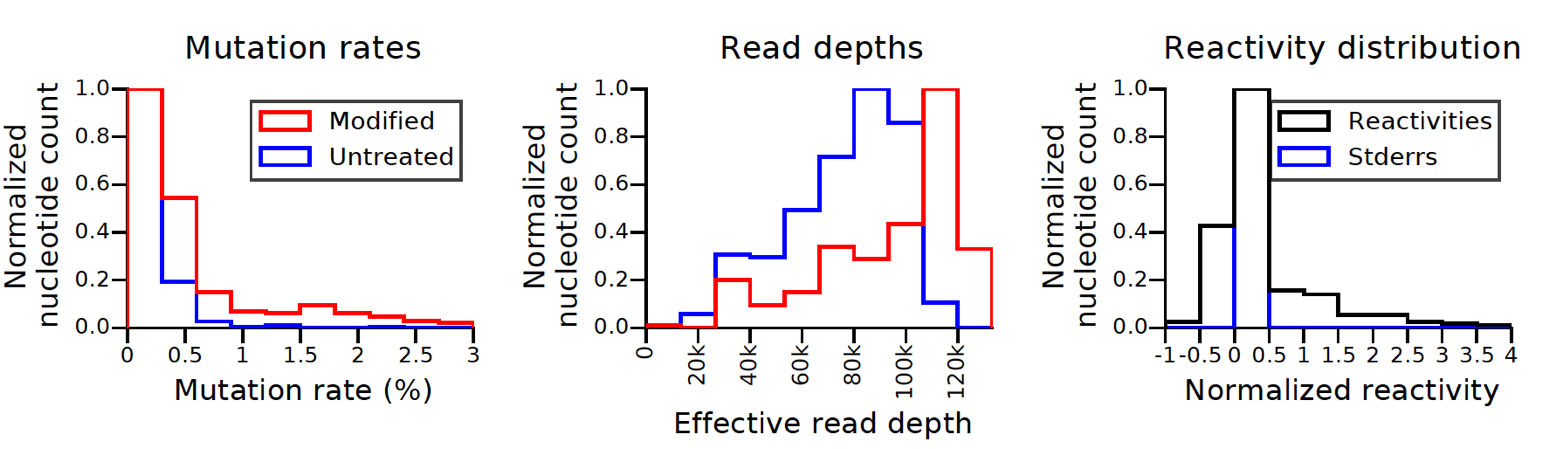


**Figure S6.** The mutation rates, read depths, and reactivity distribution of the *in vivo*, in-virion and *in vitro* DMS-MaPseqs using the ShapeMapper2 pipeline.

**Table S1.** The normalized DMS activities (ΔnDMS) and standard errors in the 3’ UTR under different DMS settings. DMS activities for G, U and the primer-binding regions are annotated as -999 (meaning unavailable data in SuperFold). The significantly higher or lower ΔnDMS activities in the in-virion and *in vitro* DMS-MaPseqs compared to the *in vivo* minigene DMS-MaPseq are highlighted in red and green, respectively (> 95% confidence in *Z*-factor test and > 1.5-fold ΔnDMS change).

| Nt# | Base | *in vivo* minigene | | in-virion | | *in vitro* | | |  |
| --- | --- | --- | --- | --- | --- | --- | --- | --- | --- |
|  |  | ΔnDMS | Std. error | ΔnDMS | Std. error | | ΔnDMS | Std. error | |
| 1 | A | 0 | 0 | 0 | 0 | | 0 | 0 | |
| 2 | C | 0 | 0 | 0 | 0 | | 0 | 0 | |
| 3 | U | -999 | 0 | -999 | 0 | | -999 | 0 | |
| 4 | C | 0.56797 | 0.002733 | 0.088938 | 0.000706 | | 0.27787 | 0.01725 | |
| 5 | A | 0.566271 | 0.002733 | 0.157787 | 0.000891 | | 0.229433 | 0.017437 | |
| 6 | U | -999 | 0 | -999 | 0 | | -999 | 0 | |
| 7 | G | -999 | 0 | -999 | 0 | | -999 | 0 | |
| 8 | C | 0.259719 | 0.001994 | 0.902901 | 0.00208 | | 1.205666 | 0.033249 | |
| 9 | A | 0.50666 | 0.002512 | 0.920986 | 0.002005 | | 1.342476 | 0.034749 | |
| 10 | G | -999 | 0 | -999 | 0 | | -999 | 0 | |
| 11 | A | 0.91249 | 0.003767 | 1.022847 | 0.002228 | | 1.488536 | 0.038312 | |
| 12 | C | 0.369856 | 0.002438 | 3.551854 | 0.004122 | | 1.183229 | 0.033374 | |
| 13 | C | 0.273976 | 0.001921 | 0.313234 | 0.001188 | | 0.765549 | 0.025875 | |
| 14 | A | 1.263657 | 0.003915 | 1.020471 | 0.00208 | | 2.196836 | 0.043999 | |
| 15 | C | 0.785068 | 0.003029 | 0.103978 | 0.000706 | | 0.453617 | 0.019875 | |
| 16 | A | 0.651219 | 0.002733 | 0.24342 | 0.001003 | | 0.508054 | 0.021437 | |
| 17 | C | 0.63748 | 0.002733 | 0.106615 | 0.000706 | | 0.633551 | 0.022812 | |
| 18 | A | 0.675743 | 0.002733 | 0.22333 | 0.000966 | | 0.497366 | 0.020937 | |
| 19 | A | 0.584738 | 0.002585 | 0.218614 | 0.000966 | | 0.375368 | 0.020062 | |
| 20 | G | -999 | 0 | -999 | 0 | | -999 | 0 | |
| 21 | G | -999 | 0 | -999 | 0 | | -999 | 0 | |
| 22 | C | 0.525127 | 0.002364 | 0.70668 | 0.001671 | | 0.807986 | 0.02525 | |
| 23 | A | 0.811143 | 0.002955 | 1.007585 | 0.001894 | | 1.094918 | 0.028312 | |
| 24 | G | -999 | 0 | -999 | 0 | | -999 | 0 | |
| 25 | A | 1.371061 | 0.003841 | 1.840189 | 0.002674 | | 2.013277 | 0.039687 | |
| 26 | U | -999 | 0 | -999 | 0 | | -999 | 0 | |
| 27 | G | -999 | 0 | -999 | 0 | | -999 | 0 | |
| 28 | G | -999 | 0 | -999 | 0 | | -999 | 0 | |
| 29 | G | -999 | 0 | -999 | 0 | | -999 | 0 | |
| 30 | C | 0.023195 | 0.001403 | 0.092392 | 0.000668 | | -0.081061 | 0.013312 | |
| 31 | U | -999 | 0 | -999 | 0 | | -999 | 0 | |
| 32 | A | 0.183709 | 0.001477 | 0.254821 | 0.000966 | | 0.015062 | 0.0105 | |
| 33 | U | -999 | 0 | -999 | 0 | | -999 | 0 | |
| 34 | A | 0.243099 | 0.001699 | 0.294741 | 0.00104 | | 0.069999 | 0.008562 | |
| 35 | U | -999 | 0 | -999 | 0 | | -999 | 0 | |
| 36 | A | 0.444906 | 0.002216 | 0.395525 | 0.001225 | | 0.284557 | 0.014875 | |
| 37 | A | 1.392261 | 0.003915 | 1.102502 | 0.002042 | | 1.424975 | 0.033437 | |
| 38 | A | 1.225616 | 0.003693 | 0.989686 | 0.001931 | | 1.02092 | 0.028375 | |
| 39 | C | 0.311944 | 0.001994 | 0.267447 | 0.001077 | | 0.269245 | 0.014687 | |
| 40 | G | -999 | 0 | -999 | 0 | | -999 | 0 | |
| 41 | U | -999 | 0 | -999 | 0 | | -999 | 0 | |
| 42 | U | -999 | 0 | -999 | 0 | | -999 | 0 | |
| 43 | U | -999 | 0 | -999 | 0 | | -999 | 0 | |
| 44 | U | -999 | 0 | -999 | 0 | | -999 | 0 | |
| 45 | C | 0.24731 | 0.001847 | 0.237144 | 0.001003 | | 0.242371 | 0.013375 | |
| 46 | G | -999 | 0 | -999 | 0 | | -999 | 0 | |
| 47 | C | 1.846327 | 0.004654 | 0.907172 | 0.001968 | | 2.331897 | 0.042312 | |
| 48 | U | -999 | 0 | -999 | 0 | | -999 | 0 | |
| 49 | U | -999 | 0 | -999 | 0 | | -999 | 0 | |
| 50 | U | -999 | 0 | -999 | 0 | | -999 | 0 | |
| 51 | U | -999 | 0 | -999 | 0 | | -999 | 0 | |
| 52 | C | 2.005512 | 0.004875 | 0.898259 | 0.001894 | | 3.340129 | 0.045874 | |
| 53 | C | 0.258833 | 0.002142 | 0.206211 | 0.000966 | | 0.320057 | 0.015875 | |
| 54 | G | -999 | 0 | -999 | 0 | | -999 | 0 | |
| 55 | U | -999 | 0 | -999 | 0 | | -999 | 0 | |
| 56 | U | -999 | 0 | -999 | 0 | | -999 | 0 | |
| 57 | U | -999 | 0 | -999 | 0 | | -999 | 0 | |
| 58 | A | 0.337355 | 0.002068 | 0.356645 | 0.001188 | | 0.212684 | 0.0125 | |
| 59 | C | 0.277743 | 0.001994 | 0.215457 | 0.000966 | | 0.189934 | 0.012625 | |
| 60 | G | -999 | 0 | -999 | 0 | | -999 | 0 | |
| 61 | A | 2.426855 | 0.005245 | 1.769707 | 0.002562 | | 2.1229 | 0.036562 | |
| 62 | U | -999 | 0 | -999 | 0 | | -999 | 0 | |
| 63 | A | 0.324945 | 0.001994 | 0.304136 | 0.001077 | | 0.093998 | 0.00975 | |
| 64 | U | -999 | 0 | -999 | 0 | | -999 | 0 | |
| 65 | A | 0.328269 | 0.001994 | 0.304173 | 0.001114 | | 0.092686 | 0.010187 | |
| 66 | U | -999 | 0 | -999 | 0 | | -999 | 0 | |
| 67 | A | 0.309876 | 0.001994 | 0.330316 | 0.001151 | | 0.092186 | 0.011312 | |
| 68 | G | -999 | 0 | -999 | 0 | | -999 | 0 | |
| 69 | U | -999 | 0 | -999 | 0 | | -999 | 0 | |
| 70 | C | 0.076527 | 0.001182 | 0.066286 | 0.000594 | | 0.029062 | 0.008937 | |
| 71 | U | -999 | 0 | -999 | 0 | | -999 | 0 | |
| 72 | A | 0.787431 | 0.002955 | 0.34673 | 0.001151 | | 0.951358 | 0.024187 | |
| 73 | C | 0.559992 | 0.002438 | 0.289393 | 0.00104 | | 0.323369 | 0.014187 | |
| 74 | U | -999 | 0 | -999 | 0 | | -999 | 0 | |
| 75 | C | 0.205205 | 0.001625 | 0.09967 | 0.000668 | | 0.098561 | 0.010125 | |
| 76 | U | -999 | 0 | -999 | 0 | | -999 | 0 | |
| 77 | U | -999 | 0 | -999 | 0 | | -999 | 0 | |
| 78 | G | -999 | 0 | -999 | 0 | | -999 | 0 | |
| 79 | U | -999 | 0 | -999 | 0 | | -999 | 0 | |
| 80 | G | -999 | 0 | -999 | 0 | | -999 | 0 | |
| 81 | C | 0.613916 | 0.002807 | 0.242826 | 0.001114 | | 0.827735 | 0.02725 | |
| 82 | A | 0.894097 | 0.003324 | 1.110672 | 0.002117 | | 1.004232 | 0.025375 | |
| 83 | G | -999 | 0 | -999 | 0 | | -999 | 0 | |
| 84 | A | 1.383619 | 0.003915 | 1.268867 | 0.002191 | | 1.83928 | 0.032812 | |
| 85 | A | 1.062441 | 0.003472 | 1.252156 | 0.002191 | | 1.067044 | 0.025187 | |
| 86 | U | -999 | 0 | -999 | 0 | | -999 | 0 | |
| 87 | G | -999 | 0 | -999 | 0 | | -999 | 0 | |
| 88 | A | 2.073397 | 0.004801 | 1.459073 | 0.002377 | | 3.688998 | 0.046562 | |
| 89 | A | 1.119911 | 0.00362 | 1.395386 | 0.00234 | | 0.721112 | 0.022062 | |
| 90 | U | -999 | 0 | -999 | 0 | | -999 | 0 | |
| 91 | U | -999 | 0 | -999 | 0 | | -999 | 0 | |
| 92 | C | 0.466476 | 0.002364 | 0.944307 | 0.001931 | | 0.376493 | 0.015937 | |
| 93 | U | -999 | 0 | -999 | 0 | | -999 | 0 | |
| 94 | C | 0.595744 | 0.002733 | 1.560228 | 0.002488 | | 0.535428 | 0.018875 | |
| 95 | G | -999 | 0 | -999 | 0 | | -999 | 0 | |
| 96 | U | -999 | 0 | -999 | 0 | | -999 | 0 | |
| 97 | A | 0.36439 | 0.002068 | 0.871856 | 0.00182 | | 0.201809 | 0.01275 | |
| 98 | A | 0.320365 | 0.001921 | 0.419997 | 0.001263 | | 0.27762 | 0.014 | |
| 99 | C | 0.205796 | 0.001551 | 0.197373 | 0.000891 | | 0.097498 | 0.009062 | |
| 100 | U | -999 | 0 | -999 | 0 | | -999 | 0 | |
| 101 | A | 0.292591 | 0.001847 | 0.297712 | 0.001077 | | 0.169997 | 0.011937 | |
| 102 | C | 0.207421 | 0.001551 | 0.170933 | 0.000854 | | 0.123748 | 0.009812 | |
| 103 | A | 0.290596 | 0.001847 | 0.32326 | 0.001114 | | 0.160372 | 0.012125 | |
| 104 | U | -999 | 0 | -999 | 0 | | -999 | 0 | |
| 105 | A | 0.98525 | 0.00362 | 1.088836 | 0.002042 | | 1.012232 | 0.025437 | |
| 106 | G | -999 | 0 | -999 | 0 | | -999 | 0 | |
| 107 | C | 0.760913 | 0.002955 | 0.793984 | 0.001782 | | 0.904797 | 0.02475 | |
| 108 | A | 1.377192 | 0.003915 | 1.236077 | 0.002191 | | 0.99042 | 0.026312 | |
| 109 | C | 1.391744 | 0.003989 | 1.216544 | 0.002265 | | 1.282165 | 0.028437 | |
| 110 | A | 1.272817 | 0.003767 | 1.039298 | 0.002005 | | 1.01792 | 0.0255 | |
| 111 | A | 1.31928 | 0.003767 | 1.191478 | 0.002117 | | 1.00792 | 0.024937 | |
| 112 | G | -999 | 0 | -999 | 0 | | -999 | 0 | |
| 113 | U | -999 | 0 | -999 | 0 | | -999 | 0 | |
| 114 | A | 1.052691 | 0.003398 | 0.987309 | 0.001968 | | 0.816423 | 0.022375 | |
| 115 | G | -999 | 0 | -999 | 0 | | -999 | 0 | |
| 116 | A | 0.421416 | 0.002142 | 0.450819 | 0.0013 | | 0.29112 | 0.01475 | |
| 117 | U | -999 | 0 | -999 | 0 | | -999 | 0 | |
| 118 | G | -999 | 0 | -999 | 0 | | -999 | 0 | |
| 119 | U | -999 | 0 | -999 | 0 | | -999 | 0 | |
| 120 | A | 0.264373 | 0.001773 | 0.354788 | 0.001188 | | 0.068061 | 0.010125 | |
| 121 | G | -999 | 0 | -999 | 0 | | -999 | 0 | |
| 122 | U | -999 | 0 | -999 | 0 | | -999 | 0 | |
| 123 | U | -999 | 0 | -999 | 0 | | -999 | 0 | |
| 124 | A | 0.714672 | 0.002881 | 0.838398 | 0.001857 | | 0.364369 | 0.015375 | |
| 125 | A | 0.920024 | 0.00325 | 0.863241 | 0.001857 | | 0.695675 | 0.020625 | |
| 126 | C | 0.808262 | 0.002955 | 1.070046 | 0.00208 | | 0.285682 | 0.014625 | |
| 127 | U | -999 | 0 | -999 | 0 | | -999 | 0 | |
| 128 | U | -999 | 0 | -999 | 0 | | -999 | 0 | |
| 129 | U | -999 | 0 | -999 | 0 | | -999 | 0 | |
| 130 | A | 0.740673 | 0.003029 | 1.404818 | 0.002488 | | 0.384243 | 0.016125 | |
| 131 | A | 0.569226 | 0.002512 | 1.314692 | 0.002265 | | 0.30787 | 0.013875 | |
| 132 | U | -999 | 0 | -999 | 0 | | -999 | 0 | |
| 133 | C | 0.47172 | 0.00229 | 1.29293 | 0.002265 | | 0.294245 | 0.013187 | |
| 134 | U | -999 | 0 | -999 | 0 | | -999 | 0 | |
| 135 | C | 0.399994 | 0.002142 | 0.359356 | 0.001263 | | 0.205809 | 0.011812 | |
| 136 | A | 0.392312 | 0.002142 | 0.43214 | 0.001337 | | 0.349119 | 0.016687 | |
| 137 | C | 0.536133 | 0.002512 | 0.41127 | 0.001337 | | 0.282058 | 0.013125 | |
| 138 | A | 0.678181 | 0.002807 | 0.585805 | 0.001597 | | 0.656551 | 0.020312 | |
| 139 | U | -999 | 0 | -999 | 0 | | -999 | 0 | |
| 140 | A | 0.649298 | 0.002807 | 0.558474 | 0.00156 | | 0.464429 | 0.017437 | |
| 141 | G | -999 | 0 | -999 | 0 | | -999 | 0 | |
| 142 | C | 0.592346 | 0.002659 | 0.419589 | 0.001374 | | 0.28362 | 0.013812 | |
| 143 | A | 0.88309 | 0.003176 | 0.644813 | 0.001634 | | 0.771361 | 0.021875 | |
| 144 | A | 1.240167 | 0.003693 | 0.663269 | 0.001671 | | 0.758487 | 0.020375 | |
| 145 | U | -999 | 0 | -999 | 0 | | -999 | 0 | |
| 146 | C | 0.633712 | 0.002659 | 0.525981 | 0.001523 | | 0.14206 | 0.013187 | |
| 147 | U | -999 | 0 | -999 | 0 | | -999 | 0 | |
| 148 | U | -999 | 0 | -999 | 0 | | -999 | 0 | |
| 149 | U | -999 | 0 | -999 | 0 | | -999 | 0 | |
| 150 | A | 0.77901 | 0.003176 | 1.392564 | 0.002562 | | 0.417305 | 0.016812 | |
| 151 | A | 0.748872 | 0.002881 | 1.283832 | 0.002302 | | 0.44143 | 0.016187 | |
| 152 | U | -999 | 0 | -999 | 0 | | -999 | 0 | |
| 153 | C | 0.81528 | 0.003102 | 1.220888 | 0.00234 | | 0.803673 | 0.020937 | |
| 154 | A | 0.837735 | 0.003176 | 1.595321 | 0.002599 | | 0.623552 | 0.019687 | |
| 155 | G | -999 | 0 | -999 | 0 | | -999 | 0 | |
| 156 | U | -999 | 0 | -999 | 0 | | -999 | 0 | |
| 157 | G | -999 | 0 | -999 | 0 | | -999 | 0 | |
| 158 | U | -999 | 0 | -999 | 0 | | -999 | 0 | |
| 159 | G | -999 | 0 | -999 | 0 | | -999 | 0 | |
| 160 | U | -999 | 0 | -999 | 0 | | -999 | 0 | |
| 161 | A | 1.734196 | 0.004284 | 1.427879 | 0.002377 | | 2.885262 | 0.038687 | |
| 162 | A | 0.400733 | 0.002142 | 0.534559 | 0.001448 | | 0.329619 | 0.01575 | |
| 163 | C | 0.253293 | 0.001773 | 0.199415 | 0.000966 | | 0.112873 | 0.01225 | |
| 164 | A | 0.251077 | 0.001773 | 0.255303 | 0.00104 | | 0.129435 | 0.010687 | |
| 165 | U | -999 | 0 | -999 | 0 | | -999 | 0 | |
| 166 | U | -999 | 0 | -999 | 0 | | -999 | 0 | |
| 167 | A | 0.240144 | 0.001847 | 0.250327 | 0.001077 | | 0.175122 | 0.012625 | |
| 168 | G | -999 | 0 | -999 | 0 | | -999 | 0 | |
| 169 | G | -999 | 0 | -999 | 0 | | -999 | 0 | |
| 170 | G | -999 | 0 | -999 | 0 | | -999 | 0 | |
| 171 | A | 0.807597 | 0.003324 | 0.590076 | 0.001708 | | 1.120355 | 0.026687 | |
| 172 | G | -999 | 0 | -999 | 0 | | -999 | 0 | |
| 173 | G | -999 | 0 | -999 | 0 | | -999 | 0 | |
| 174 | A | 1.816854 | 0.004654 | 1.072348 | 0.002191 | | 3.205881 | 0.041812 | |
| 175 | C | 0.970254 | 0.003472 | 0.962057 | 0.00208 | | 0.925421 | 0.023 | |
| 176 | U | -999 | 0 | -999 | 0 | | -999 | 0 | |
| 177 | U | -999 | 0 | -999 | 0 | | -999 | 0 | |
| 178 | G | -999 | 0 | -999 | 0 | | -999 | 0 | |
| 179 | A | 1.018638 | 0.003472 | 0.844785 | 0.001968 | | 1.322164 | 0.028437 | |
| 180 | A | 0.945213 | 0.003324 | 0.857151 | 0.002005 | | 1.360601 | 0.027187 | |
| 181 | A | 0.745401 | 0.003029 | 0.718006 | 0.001857 | | 1.053669 | 0.025187 | |
| 182 | G | -999 | 0 | -999 | 0 | | -999 | 0 | |
| 183 | A | 0.541525 | 0.002585 | 0.447737 | 0.001448 | | 0.729862 | 0.022875 | |
| 184 | G | -999 | 0 | -999 | 0 | | -999 | 0 | |
| 185 | C | 0.059094 | 0.001182 | 0.05897 | 0.000631 | | 0.167997 | 0.012625 | |
| 186 | C | 0.015512 | 0.001182 | 0.050652 | 0.000594 | | 0.146247 | 0.011625 | |
| 187 | A | 1.499961 | 0.004137 | 1.241907 | 0.002377 | | 2.916574 | 0.041124 | |
| 188 | C | 0.113978 | 0.001256 | 0.12767 | 0.000817 | | 0.217809 | 0.013062 | |
| 189 | C | 0.112648 | 0.001256 | 0.105909 | 0.00078 | | 0.141435 | 0.010812 | |
| 190 | A | 1.127667 | 0.003546 | 0.994773 | 0.002117 | | 2.167024 | 0.034874 | |
| 191 | C | 0.255435 | 0.001773 | 0.224741 | 0.00104 | | 0.212059 | 0.0115 | |
| 192 | A | 0.217172 | 0.001847 | 0.221139 | 0.001114 | | 0.056749 | 0.01275 | |
| 193 | U | -999 | 0 | -999 | 0 | | -999 | 0 | |
| 194 | U | -999 | 0 | -999 | 0 | | -999 | 0 | |
| 195 | U | -999 | 0 | -999 | 0 | | -999 | 0 | |
| 196 | U | -999 | 0 | -999 | 0 | | -999 | 0 | |
| 197 | C | 0.144338 | 0.001551 | 0.115081 | 0.00078 | | 0.148497 | 0.0105 | |
| 198 | A | 0.351685 | 0.002142 | 0.232205 | 0.001077 | | 0.396556 | 0.016937 | |
| 199 | C | 0.080516 | 0.001182 | 0.10357 | 0.001485 | | 0.136373 | 0.010687 | |
| 200 | C | 0.356929 | 0.002142 | 0.230126 | 0.001077 | | 0.219059 | 0.0125 | |
| 201 | G | -999 | 0 | -999 | 0 | | -999 | 0 | |
| 202 | A | 1.589415 | 0.004284 | 1.162884 | 0.00234 | | 2.650641 | 0.038562 | |
| 203 | G | -999 | 0 | -999 | 0 | | -999 | 0 | |
| 204 | G | -999 | 0 | -999 | 0 | | -999 | 0 | |
| 205 | C | 0.456208 | 0.002364 | 0.479116 | 0.001634 | | 0.266245 | 0.01675 | |
| 206 | C | 0.093591 | 0.001256 | 0.114413 | 0.000817 | | 0.146122 | 0.011875 | |
| 207 | A | 0.595006 | 0.002659 | 0.469535 | 0.001485 | | 3.689748 | 0.046312 | |
| 208 | C | 0.231428 | 0.001847 | 0.223701 | 0.001188 | | 0.508991 | 0.01825 | |
| 209 | G | -999 | 0 | -999 | 0 | | -999 | 0 | |
| 210 | C | 0.311427 | 0.001994 | 0.240709 | 0.001114 | | 0.488491 | 0.017437 | |
| 211 | G | -999 | 0 | -999 | 0 | | -999 | 0 | |
| 212 | G | -999 | 0 | -999 | 0 | | -999 | 0 | |
| 213 | A | 1.405558 | 0.003915 | 1.254384 | 0.00234 | | 1.537535 | 0.028874 | |
| 214 | G | -999 | 0 | -999 | 0 | | -999 | 0 | |
| 215 | U | -999 | 0 | -999 | 0 | | -999 | 0 | |
| 216 | A | 1.214905 | 0.003693 | 1.022179 | 0.002154 | | 1.063919 | 0.024562 | |
| 217 | C | 1.899955 | 0.004654 | 1.42346 | 0.002599 | | 1.324477 | 0.027312 | |
| 218 | G | -999 | 0 | -999 | 0 | | -999 | 0 | |
| 219 | A | 1.129144 | 0.003693 | 0.953888 | 0.002117 | | 1.287977 | 0.028 | |
| 220 | U | -999 | 0 | -999 | 0 | | -999 | 0 | |
| 221 | C | 0.3491 | 0.002364 | 0.371685 | 0.001485 | | 0.559865 | 0.01925 | |
| 222 | G | -999 | 0 | -999 | 0 | | -999 | 0 | |
| 223 | A | 0.829832 | 0.00325 | 0.716409 | 0.001968 | | 1.814531 | 0.032124 | |
| 224 | G | -999 | 0 | -999 | 0 | | -999 | 0 | |
| 225 | U | -999 | 0 | -999 | 0 | | -999 | 0 | |
| 226 | G | -999 | 0 | -999 | 0 | | -999 | 0 | |
| 227 | U | -999 | 0 | -999 | 0 | | -999 | 0 | |
| 228 | A | 0.883607 | 0.003472 | 0.814371 | 0.002117 | | 0.236433 | 0.014312 | |
| 229 | C | 0.520547 | 0.002733 | 0.442538 | 0.001634 | | 0.291307 | 0.014562 | |
| 230 | A | 0.929701 | 0.003546 | 0.564452 | 0.001782 | | 0.592427 | 0.020187 | |
| 231 | G | -999 | 0 | -999 | 0 | | -999 | 0 | |
| 232 | U | -999 | 0 | -999 | 0 | | -999 | 0 | |
| 233 | G | -999 | 0 | -999 | 0 | | -999 | 0 | |
| 234 | A | 0.347327 | 0.002364 | 0.311711 | 0.001448 | | 0.339807 | 0.017062 | |
| 235 | A | 0.309802 | 0.002216 | 0.302279 | 0.001411 | | 0.345119 | 0.017937 | |
| 236 | C | 2.12732 | 0.005614 | 2.021631 | 0.003602 | | 2.109838 | 0.036312 | |
| 237 | A | 0.394898 | 0.002512 | 0.367117 | 0.001523 | | 0.250308 | 0.01425 | |
| 238 | A | 0.319996 | 0.002216 | 0.301611 | 0.001411 | | 0.244933 | 0.014875 | |
| 239 | U | -999 | 0 | -999 | 0 | | -999 | 0 | |
| 240 | G | -999 | 0 | -999 | 0 | | -999 | 0 | |
| 241 | C | 1.689801 | 0.005097 | 1.174433 | 0.002897 | | 1.615222 | 0.032937 | |
| 242 | U | -999 | 0 | -999 | 0 | | -999 | 0 | |
| 243 | A | 1.582693 | 0.005023 | 1.242018 | 0.002897 | | 1.411663 | 0.031874 | |
| 244 | G | -999 | 0 | -999 | 0 | | -999 | 0 | |
| 245 | G | -999 | 0 | -999 | 0 | | -999 | 0 | |
| 246 | G | -999 | 0 | -999 | 0 | | -999 | 0 | |
| 247 | A | 1.422621 | 0.005097 | 1.148364 | 0.003008 | | 1.760032 | 0.035812 | |
| 248 | G | -999 | 0 | -999 | 0 | | -999 | 0 | |
| 249 | A | 1.783096 | 0.005762 | 1.233626 | 0.003119 | | 2.322709 | 0.042312 | |
| 250 | G | -999 | 0 | -999 | 0 | | -999 | 0 | |
| 251 | C | 0.169674 | 0.002068 | 0.162466 | 0.001263 | | 0.115685 | 0.012187 | |
| 252 | U | -999 | 0 | -999 | 0 | | -999 | 0 | |
| 253 | G | -999 | 0 | -999 | 0 | | -999 | 0 | |
| 254 | C | 0.397483 | 0.002807 | 0.537492 | 0.00208 | | 0.412993 | 0.022187 | |
| 255 | C | 0.171964 | 0.001994 | 0.302353 | 0.001597 | | 0.065311 | 0.012687 | |
| 256 | U | -999 | 0 | -999 | 0 | | -999 | 0 | |
| 257 | A | 1.559055 | 0.005245 | 1.273026 | 0.003156 | | 1.058231 | 0.029249 | |
| 258 | U | -999 | 0 | -999 | 0 | | -999 | 0 | |
| 259 | A | 1.471005 | 0.005171 | 1.071866 | 0.002934 | | 1.503536 | 0.034124 | |
| 260 | U | -999 | 0 | -999 | 0 | | -999 | 0 | |
| 261 | G | -999 | 0 | -999 | 0 | | -999 | 0 | |
| 262 | G | -999 | 0 | -999 | 0 | | -999 | 0 | |
| 263 | A | 0.75552 | 0.004063 | 0.927039 | 0.002971 | | 0.847798 | 0.027937 | |
| 264 | A | 1.497671 | 0.005614 | 1.073945 | 0.003194 | | 3.200569 | 0.052874 | |
| 265 | G | -999 | 0 | -999 | 0 | | -999 | 0 | |
| 266 | A | 1.748526 | 0.006131 | 1.365195 | 0.003714 | | 2.770826 | 0.049874 | |
| 267 | G | -999 | 0 | -999 | 0 | | -999 | 0 | |
| 268 | C | 0.062418 | 0.001625 | 0.038695 | 0.000854 | | 0.039124 | 0.016437 | |
| 269 | C | 0.018467 | 0.001182 | 0.021018 | 0.000706 | | 0.124873 | 0.015687 | |
| 270 | C | 0.084726 | 0.001773 | 0.035761 | 0.000854 | | -0.165747 | 0.020312 | |
| 271 | U | -999 | 0 | -999 | 0 | | -999 | 0 | |
| 272 | A | 0.253884 | 0.002512 | 0.277547 | 0.001745 | | 0.251246 | 0.016687 | |
| 273 | A | 0.269839 | 0.002512 | 0.24977 | 0.001671 | | 0.207934 | 0.01525 | |
| 274 | U | -999 | 0 | -999 | 0 | | -999 | 0 | |
| 275 | G | -999 | 0 | -999 | 0 | | -999 | 0 | |
| 276 | U | -999 | 0 | -999 | 0 | | -999 | 0 | |
| 277 | G | -999 | 0 | -999 | 0 | | -999 | 0 | |
| 278 | U | -999 | 0 | -999 | 0 | | -999 | 0 | |
| 279 | A | 1.364192 | 0.005909 | 0.636829 | 0.002859 | | 2.124525 | 0.045812 | |
| 280 | A | 0.907762 | 0.004801 | 0.574442 | 0.002711 | | 0.624677 | 0.025687 | |
| 281 | A | 1.071306 | 0.005171 | 0.899819 | 0.003379 | | 0.676801 | 0.025687 | |
| 282 | A | 0.772879 | 0.004506 | 0.932052 | 0.003416 | | 0.352744 | 0.020187 | |
| 283 | U | -999 | 0 | -999 | 0 | | -999 | 0 | |
| 284 | U | -999 | 0 | -999 | 0 | | -999 | 0 | |
| 285 | A | 0.959396 | 0.005097 | 0.967887 | 0.003639 | | 0.68305 | 0.027687 | |
| 286 | A | 0.960061 | 0.005171 | 1.310087 | 0.004196 | | 0.208621 | 0.026375 | |
| 287 | U | -999 | 0 | -999 | 0 | | -999 | 0 | |
| 288 | U | -999 | 0 | -999 | 0 | | -999 | 0 | |
| 289 | U | -999 | 0 | -999 | 0 | | -999 | 0 | |
| 290 | U | -999 | 0 | -999 | 0 | | -999 | 0 | |
| 291 | A | 0.986579 | 0.005245 | 1.02515 | 0.003788 | | 0.425993 | 0.022312 | |
| 292 | G | -999 | 0 | -999 | 0 | | -999 | 0 | |
| 293 | U | -999 | 0 | -999 | 0 | | -999 | 0 | |
| 294 | A | 1.485926 | 0.006205 | 2.329183 | 0.00557 | | 1.241041 | 0.040124 | |
| 295 | G | -999 | 0 | -999 | 0 | | -999 | 0 | |
| 296 | U | -999 | 0 | -999 | 0 | | -999 | 0 | |
| 297 | G | -999 | 0 | -999 | 0 | | -999 | 0 | |
| 298 | C | 0.522911 | 0.003989 | 0.586139 | 0.003082 | | 0.133935 | 0.018437 | |
| 299 | U | -999 | 0 | -999 | 0 | | -999 | 0 | |
| 300 | A | 1.414791 | 0.006353 | 1.139637 | 0.004233 | | 0.938858 | 0.038249 | |
| 301 | U | -999 | 0 | -999 | 0 | | -999 | 0 | |
| 302 | C | 1.680863 | 0.007017 | -999 | 0 | | 1.805031 | 0.056437 | |
| 303 | C | 1.061629 | 0.006057 | -999 | 0 | | 1.769406 | 0.055749 | |
| 304 | C | 2.411269 | 0.009529 | -999 | 0 | | 2.101526 | 0.059311 | |
| 305 | C | 2.729639 | 0.010489 | -999 | 0 | | 1.950528 | 0.056874 | |
| 306 | A | 1.527218 | 0.007756 | -999 | 0 | | 1.116668 | 0.042874 | |
| 307 | U | -999 | 0 | -999 | 0 | | -999 | 0 | |
| 308 | G | -999 | 0 | -999 | 0 | | -999 | 0 | |
| 309 | U | -999 | 0 | -999 | 0 | | -999 | 0 | |
| 310 | G | -999 | 0 | -999 | 0 | | -999 | 0 | |
| 311 | A | 0.648708 | 0.005909 | -999 | 0 | | -999 | 0 | |
| 312 | U | -999 | 0 | -999 | 0 | | -999 | 0 | |
| 313 | U | -999 | 0 | -999 | 0 | | -999 | 0 | |
| 314 | U | -999 | 0 | -999 | 0 | | -999 | 0 | |
| 315 | U | -999 | 0 | -999 | 0 | | -999 | 0 | |
| 316 | A | 1.051066 | 0.007313 | -999 | 0 | | -999 | 0 | |
| 317 | A | 1.121388 | 0.007682 | -999 | 0 | | -999 | 0 | |
| 318 | U | -999 | 0 | -999 | 0 | | -999 | 0 | |
| 319 | A | -999 | 0 | -999 | 0 | | -999 | 0 | |
| 320 | G | -999 | 0 | -999 | 0 | | -999 | 0 | |
| 321 | C | -999 | 0 | -999 | 0 | | -999 | 0 | |
| 322 | U | -999 | 0 | -999 | 0 | | -999 | 0 | |
| 323 | U | -999 | 0 | -999 | 0 | | -999 | 0 | |
| 324 | C | -999 | 0 | -999 | 0 | | -999 | 0 | |
| 325 | U | -999 | 0 | -999 | 0 | | -999 | 0 | |
| 326 | U | -999 | 0 | -999 | 0 | | -999 | 0 | |
| 327 | A | -999 | 0 | -999 | 0 | | -999 | 0 | |

The SARS-CoV-2 minigene plasmid (pUC57-COVID-MG) sequence*

*CAGGAAACAGCTATGAC*CATGATTACGCCAAGCTTGCATGCAGGCCTCTGCAGTCGACGGGCCCGGGATCCGATCCAATTTAATACGACTCACTATAGGATTAAAGGTTTATACCTTCCCAGGTAACAAACCAACCAACTTTCGATCTCTTGTAGATCTGTTCTCTAAACGAACTTTAAAATCTGTGTGGCTGTCACTCGGCTGCATGCTTAGTGCACTCACGCAGTATAATTAATAACTAATTACTGTCGTTGACAGGACACGAGTAACTCGTCTATCTTCTGCAGGCTGCTTACGGTTTCGTCCGTGTTGCAGCCGATCATCAGCACATCTAGGTTTCGTCCGGGTGTGACCGAAAGGTAAGATGGAGAGCCTTGTCCCTGGTTTCAACGAGAAAACACACGTCCAACTCAGTTTGCCTGTTTTACAGGTTCGCGACGTGCTCGTACGTGGCTTTGGAGACTCCGTGGAGGAGGTCTTATCAGAGGCACGTCAAGGAGTCAAAGTTCTGTTTGCCCTGATCTGCATCGCTGTGGCCGAGGCCAAGCCCACCGAGAACAACGAAGACTTCAACATCGTGGCCGTGGCCAGCAACTTCGCGACCACGGATCTCGATGCTGACCGCGGGAAGTTGCCCGGCAAGAAGCTGCCGCTGGAGGTGCTCAAAGAGATGGAAGCCAATGCCCGGAAAGCTGGCTGCACCAGGGGCTGTCTGATCTGCCTGTCCCACATCAAGTGCACGCCCAAGATGAAGAAGTTCATCCCAGGACGCTGCCACACCTACGAAGGCGACAAAGAGTCCGCACAGGGCGGCATAGGCGAGGCGATCGTCGACATTCCTGAGATTCCTGGGTTCAAGGACTTGGAGCCCATGGAGCAGTTCATCGCACAGGTCGATCTGTGTGTGGACTGCACAACTGGCTGCCTCAAAGGGCTTGCCAACGTGCAGTGTTCTGACCTGCTCAAGAAGTGGCTGCCGCAACGCTGTGCGACCTTTGCCAGCAAGATCCAGGGCCAGGTGGACAAGATCAAGGGGGCCGGTGGTGACACTCATGCAGACCACACAAGGCAGATGGGCTATATAAACGTTTTCGCTTTTCCGTTTACGATATATAGTCTACTCTTGTGCAGAATGAATTCTCGTAACTACATAGCACAAGTAGATGTAGTTAACTTTAATCTCACATAGCAATCTTTAATCAGTGTGTAACATTAGGGAGGACTTGAAAGAGCCACCACATTTTCACCGAGGCCACGCGGAGTACGATCGAGTGTACAGTGAACAATGCTAGGGAGAGCTGCCTATATGGAAGAGCCCTAATGTGTAAAATTAATTTTAGTAGTGCTATCCCCATGTGATTTTAATAGCTTCTTAGGAGAATGACAAAAAAAAAAAAAAAAAAAAAAAACTAGCATAACCCCTTGGGGCCTCTAAACGGGTCTTGAGGGGTTTTTTCCAATATCTAGATGCATTCGCGAGGTACCGAGCTCGAATTC*ACTGGCCGTCGTTTTAC*

* Insertion between the sequencing primers M13 reverse and M13 forward (italic) is shown in color for 5’ UTR, nsp1 (partial), *Gaussia* luciferase, 3’ UTR in this study, and poly(A). The T7 transcription promoter and terminator sequences are underlined.

The terminal command lines for deltaSHAPE_v1.0:

python deltaSHAPE.py invirion.map invivo-minigene.map -o virion-vs-vivo --pdf --noshow --all -s 1.5

python deltaSHAPE.py invitro.map invivo-minigene.map -o vitro-vs-vivo --pdf --noshow --all -s 1.5

The terminal command lines for DREEM analysis in BSL/SL1 RNA segments with Parallel [3]:

*In vivo minigene*

ls -1 *_mate1.fastq | cut -d_ -f1 -f2 | sort | uniq | parallel -j 2 'Run_DREEM.py [data path] [data path]/{ } { } pUC.fasta pUC 964 1070 --fastq --MAX_K 4'

>pUC.fasta

ATTAAAGGTTTATACCTTCCCAGGTAACAAACCAACCAACTTTCGATCTCTTGTAGATCTGTTCTCTAAACGAACTTTAAAATCTGTGTGGCTGTCACTCGGCTGCATGCTTAGTGCACTCACGCAGTATAATTAATAACTAATTACTGTCGTTGACAGGACACGAGTAACTCGTCTATCTTCTGCAGGCTGCTTACGGTTTCGTCCGTGTTGCAGCCGATCATCAGCACATCTAGGTTTCGTCCGGGTGTGACCGAAAGGTAAGATGGAGAGCCTTGTCCCTGGTTTCAACGAGAAAACACACGTCCAACTCAGTTTGCCTGTTTTACAGGTTCGCGACGTGCTCGTACGTGGCTTTGGAGACTCCGTGGAGGAGGTCTTATCAGAGGCACGTCAAGGAGTCAAAGTTCTGTTTGCCCTGATCTGCATCGCTGTGGCCGAGGCCAAGCCCACCGAGAACAACGAAGACTTCAACATCGTGGCCGTGGCCAGCAACTTCGCGACCACGGATCTCGATGCTGACCGCGGGAAGTTGCCCGGCAAGAAGCTGCCGCTGGAGGTGCTCAAAGAGATGGAAGCCAATGCCCGGAAAGCTGGCTGCACCAGGGGCTGTCTGATCTGCCTGTCCCACATCAAGTGCACGCCCAAGATGAAGAAGTTCATCCCAGGACGCTGCCACACCTACGAAGGCGACAAAGAGTCCGCACAGGGCGGCATAGGCGAGGCGATCGTCGACATTCCTGAGATTCCTGGGTTCAAGGACTTGGAGCCCATGGAGCAGTTCATCGCACAGGTCGATCTGTGTGTGGACTGCACAACTGGCTGCCTCAAAGGGCTTGCCAACGTGCAGTGTTCTGACCTGCTCAAGAAGTGGCTGCCGCAACGCTGTGCGACCTTTGCCAGCAAGATCCAGGGCCAGGTGGACAAGATCAAGGGGGCCGGTGGTGACACTCATGCAGACCACACAAGGCAGATGGGCTATATAAACGTTTTCGCTTTTCCGTTTACGATATATAGTCTACTCTTGTGCAGAATGAATTCTCGTAACTACATAGCACAAGTAGATGTAGTTAACTTTAATCTCACATAGCAATCTTTAATCAGTGTGTAACATTAGGGAGGACTTGAAAGAGCCACCACATTTTCACCGAGGCCACGCGGAGTACGATCGAGTGTACAGTGAACAATGCTAGGGAGAGCTGCCTATATGGAAGAGCCCTAATGTGTAAAATTAATTTTAGTAGTGCTATCCCCATGTGATTTTAATAGCTTCTTAGGAGAATGACAAAAA

*In-virion*

ls -1 *_mate1.fastq | cut -d_ -f1 -f2 | sort | uniq | parallel -j 2 'Run_DREEM.py [data path] [data path]/{ } { } COVID3UTR.fasta COVID3UTR 583 689 --fastq --MAX_K 4'

>COVID3UTR.fasta

GAGCAAAATGTCTGGTAAAGGCCAACAACAACAAGGCCAAACTGTCACTAAGAAATCTGCTGCTGAGGCTTCTAAGAAGCCTCGGCAAAAACGTACTGCCACTAAAGCATACAATGTAACACAAGCTTTCGGCAGACGTGGTCCAGAACAAACCCAAGGAAATTTTGGGGACCAGGAACTAATCAGACAAGGAACTGATTACAAACATTGGCCGCAAATTGCACAATTTGCCCCCAGCGCTTCAGCGTTCTTCGGAATGTCGCGCATTGGCATGGAAGTCACACCTTCGGGAACGTGGTTGACCTACACAGGTGCCATCAAATTGGATGACAAAGATCCAAATTTCAAAGATCAAGTCATTTTGCTGAATAAGCATATTGACGCATACAAAACATTCCCACCAACAGAGCCTAAAAAGGACAAAAAGAAGAAGGCTGATGAAACTCAAGCCTTACCGCAGAGACAGAAGAAACAGCAAACTGTGACTCTTCTTCCTGCTGCAGATTTGGATGATTTCTCCAAACAATTGCAACAATCCATGAGCAGTGCTGACTCAACTCAGGCCTAAACTCATGCAGACCACACAAGGCAGATGGGCTATATAAACGTTTTCGCTTTTCCGTTTACGATATATAGTCTACTCTTGTGCAGAATGAATTCTCGTAACTACATAGCACAAGTAGATGTAGTTAACTTTAATCTCACATAGCAATCTTTAATCAGTGTGTAACATTAGGGAGGACTTGAAAGAGCCACCACATTTTCACCGAGGCCACGCGGAGTACGATCGAGTGTACAGTGAACAATGCTAGGGAGAGCTGCCTATATGGAAGAGCCCTAATGTGTAAAATTAATTTTAGTAGTGCTATCCCCATGTGATTTTAATAGCTTCTTA

References

1. Smola, M.J.; Rice, G.M.; Busan, S.; Siegfried, N.A.; Weeks, K.M. Selective 2’-hydroxyl acylation analyzed by primer extension and mutational profiling (SHAPE-MaP) for direct, versatile and accurate RNA structure analysis. *Nat. Protoc.* **2015**, *10*, 1643–69, doi:10.1038/nprot.2015.103.

2. Tomezsko, P.J.; Corbin, V.D.A.; Gupta, P.; Swaminathan, H.; Glasgow, M.; Persad, S.; Edwards, M.D.; Mcintosh, L.; Papenfuss, A.T.; Emery, A.; et al. Determination of RNA structural diversity and its role in HIV-1 RNA splicing. *Nature* **2020**, *582*, 438–442, doi:10.1038/s41586-020-2253-5.

3. Tange, O. GNU Parallel 20200722. *Zenodo* **2020**, doi:10.5281/zenodo.3956817.

Names of the SARS-CoV-2 sequence records that are truncated in S3-B (collected and deposited in GISAID database on or before November 6, 2020). All sequence records contain a full 3’ UTR with a > 10-nt long poly(A) tail.

Vietnam/VR03-38142/2020/144-500

SouthKorea/KCDC12/2020/147-500

USA/WA3-UW1/2020/131-500

USA/WA4-UW2/2020/131-500

USA/WA9-UW6/2020/131-500

USA/WA17-UW13/2020/131-500

USA/WA-UW65/2020/132-500

USA/WA-UW40/2020/115-500

Vietnam/38142/2020/144-500

USA/WA-UW212/2020/131-500

USA/WA-UW228/2020/131-500

Malaysia/189332/2020/131-500

Malaysia/188407/2020/131-500

Malaysia/190300/2020/131-500

USA/VA-DCLS-0001/2020/131-500

USA/WA-UW345/2020/131-500

USA/WA-UW346/2020/131-500

USA/WA-UW384/2020/131-500

USA/WA-UW390/2020/116-500

USA/UT-023/2020/131-500

USA/UT-025/2020/131-500

USA/VA-DCLS-00011/2020/131-500

USA/VA-DCLS-0012/2020/131-500

USA/VA-DCLS-0042/2020/131-500

USA/VA-DCLS-0018/2020/131-500

USA/VA-DCLS-0034/2020/131-500

USA/UT-0352/2020/131-500

USA/ID-UW-4357/2020/131-500

USA/ID-UW-4427/2020/113-500

USA/ID-UW-4433/2020/131-500

USA/ID-UW-4461/2020/131-500

USA/WA-UW-2072/2020/131-500

USA/WA-UW-2142/2020/117-500

USA/WA-UW-2146/2020/131-500

SouthKorea/KCDC2003/2020/147-500

USA/VA-DCLS-0041/2020/131-500

USA/CT-UW-3845/2020/131-500

USA/ID-UW-4462/2020/131-500

USA/OR-UW-4300/2020/131-500

USA/WA-UW-3923/2020/113-500

USA/WA-UW-3935/2020/131-500

USA/WA-UW-3941/2020/113-500

USA/WA-UW-3942/2020/113-500

USA/WA-UW-4337/2020/119-500

USA/VA-DCLS-0079/2020/131-500

USA/CT-UW-5772/2020/117-500

USA/WA-UW-5879/2020/113-500

USA/WA-UW-5884/2020/113-500

USA/WA-UW-4226/2020/131-500

USA/WA-UW-4243/2020/131-500

USA/ID-UW-4255/2020/131-500

USA/WA-UW-4279/2020/131-500

USA/ID-UW-4307/2020/113-500

USA/WA-UW-4338/2020/131-500

USA/CT-UW-4343/2020/131-500

USA/CT-UW-4345/2020/117-500

USA/CT-UW-4348/2020/113-500

USA/ID-UW-4354/2020/131-500

USA/ID-UW-4402/2020/113-500

USA/OR-UW-4410/2020/115-500

USA/WA-UW-4413/2020/113-500

USA/WA-UW-6185/2020/132-500

USA/ID-UW-6349/2020/113-500

India/TG-GMC-RK100/2020/131-500

India/TG-GMC-KN443/2020/131-500

CostaRica/04/2020/191-500

Thailand/NIH-2720/2020/149-500

Thailand/NIH-2980/2020/131-500

USA/VA-DCLS-0217/2020/131-500

USA/CA-SR0193/2020/131-500

India/TG-GMC-KP1125/2020/131-500

USA/VA-DCLS-0167/2020/131-500

USA/WA-UW-4051/2020/131-500

USA/WA-UW-4058/2020/115-500

USA/WA-UW-4221/2020/117-500

USA/WA-UW-4224/2020/131-500

USA/CT-UW-4237/2020/131-500

USA/ID-UW-6467/2020/138-500

USA/WA-UW-6480/2020/113-500

USA/WA-UW-6483/2020/115-500

USA/WA-UW-6484/2020/131-500

USA/WA-UW-6485/2020/131-500

USA/WA-UW-6491/2020/113-500

USA/WA-UW-6497/2020/131-500

USA/WA-UW-6501/2020/113-500

USA/WA-UW-6547/2020/113-500

India/TG-GMC-RR1191/2020/131-500

India/TG-GMC-RK1090/2020/131-500

USA/WA-UW-4296/2020/113-500

USA/OR-UW-4409/2020/131-500

USA/WA-UW-4414/2020/131-500

Chile/Antofagasta_1/2020/159-500

Chile/Valparaiso_4/2020/60-500

Chile/Rancagua_8/2020/178-500

USA/UT-01573/2020/131-500

USA/UT-03470/2020/131-500

USA/UT-03482/2020/131-500

USA/UT-03696/2020/131-500

USA/UT-01234/2020/131-500

USA/VA-DCLS-0236/2020/131-500

CostaRica/CV-0007/2020/44-500

USA/WA-UW-3904/2020/113-500

USA/ID-UW-4376/2020/131-500

USA/WA-UW-4659/2020/131-500

USA/WA-UW-5714/2020/131-500

USA/WA-UW-5860/2020/131-500

USA/WA-UW-10027/2020/113-500

USA/WA-UW-10028/2020/113-500

USA/WA-UW-10029/2020/113-500

USA/WA-UW-10031/2020/113-500

USA/WA-UW-10039/2020/112-500

USA/WA-UW-10040/2020/113-500

USA/WA-UW-10042/2020/111-500

USA/WA-UW-10088/2020/113-500

USA/WA-UW-10089/2020/113-500

USA/WA-UW-10090/2020/131-500

USA/WA-UW-10093/2020/121-500

USA/WA-UW-10094/2020/113-500

USA/WA-UW-10102/2020/113-500

USA/WA-UW-10106/2020/125-500

USA/WA-UW-10108/2020/125-500

USA/WA-UW-10117/2020/113-500

USA/WA-UW-10118/2020/113-500

USA/WA-UW-10127/2020/113-500

USA/WA-UW-10128/2020/113-500

USA/WA-UW-10129/2020/113-500

USA/WA-UW-10130/2020/113-500

USA/WA-UW-10131/2020/131-500

USA/WA-UW-10136/2020/113-500

USA/WA-UW-10138/2020/131-500

USA/VA-DCLS-0301/2020/131-500

USA/VA-DCLS-0381/2020/131-500

USA/VA-DCLS-0387/2020/131-500

USA/VA-DCLS-0390/2020/131-500

USA/VA-DCLS-0399/2020/131-500

USA/VA-DCLS-0400/2020/131-500

USA/VA-DCLS-0321/2020/131-500

USA/WA-UW-623/2020/131-500

USA/MN-UW-628/2020/131-500

USA/CT-UW-645/2020/131-500

USA/CT-UW-647/2020/113-500

USA/CT-UW-649/2020/131-500

USA/CT-UW-650/2020/131-500

USA/CT-UW-652/2020/113-500

USA/WA-UW-669/2020/131-500

USA/WA-UW-865/2020/115-500

USA/WA-UW-896/2020/113-500

USA/VA-DCLS-0431/2020/131-500

USA/VA-DCLS-0438/2020/131-500

USA/VA-DCLS-0442/2020/131-500

USA/VA-DCLS-0450/2020/131-500

USA/VA-DCLS-0451/2020/131-500

USA/VA-DCLS-0456/2020/131-500

USA/VA-DCLS-0459/2020/131-500

USA/VA-DCLS-0460/2020/131-500

USA/VA-DCLS-0462/2020/131-500

USA/VA-DCLS-0466/2020/131-500

USA/CT-UW-2447/2020/131-500

USA/ID-UW-2047/2020/132-500

USA/UN-UW-2476/2020/131-500

USA/WA-UW-1481/2020/113-500

USA/WA-UW-1613/2020/131-500

USA/WA-UW-2056/2020/114-500

USA/WA-UW-2076/2020/131-500

USA/WA-UW-2281/2020/117-500

USA/WA-UW-2603/2020/131-500

England/OXON-AF7B3/2020/131-500

USA/CA-ALSR-0482-SAN/2020/131-500

USA/CA-ALSR-0491-SAN/2020/131-500

USA/VA-DCLS-0545/2020/131-500

USA/VA-DCLS-0563/2020/131-500

USA/ID-UW-2480/2020/113-500

USA/WA-UW-2778/2020/115-500

USA/WA-UW-2875/2020/115-500

USA/WA-UW-2929/2020/131-500

USA/WA-UW-2935/2020/131-500

USA/WA-UW-2953/2020/131-500

USA/WA-UW-2976/2020/131-500

USA/WA-UW-2983/2020/131-500

USA/WA-UW-7166/2020/131-500

USA/WA-UW-7178/2020/131-500

USA/WA-UW-7399/2020/121-500

USA/WA-UW-8150/2020/131-500

USA/MD-MDH-0037/2020/138-500

USA/CT-UW-3417/2020/121-500

USA/CT-UW-3423/2020/115-500

USA/CT-UW-3425/2020/131-500

USA/CT-UW-3470/2020/116-500

USA/ID-UW-3196/2020/131-500

USA/ID-UW-3204/2020/113-500

Italy/VEN-UniVR-5/2020/131-500

Italy/VEN-UniVR-8/2020/131-500

USA/CA-ALSR-1509/2020/131-500

USA/VA-DCLS-0636/2020/131-500

USA/VA-DCLS-0640/2020/131-500

USA/VA-DCLS-0644/2020/131-500

USA/VA-DCLS-0654/2020/131-500

India/MH-AFMC-5304/2020/133-500

India/MH-AFMC-5473/2020/134-500

India/MH-BJMC-1769/2020/133-500

India/MH-NCCS-NR1205/2020/133-500

India/MH-NCCS-NR1922/2020/132-500

India/MH-NCCS-NR2257/2020/132-500

India/MH-BJMC-4555/2020/147-500

India/MH-AFMC-5458/2020/133-500

India/MH-AFMC-4954/2020/133-500

India/MH-BJMC-1658/2020/133-500

India/MH-BJMC-965/2020/133-500

India/MH-NCCS-NR1323/2020/133-500

India/MH-NCCS-NR1553/2020/134-500

India/MH-NCCS-NR2246/2020/133-500

India/MH-NCCS-NR2260/2020/133-500

India/MH-AFMC-5280/2020/135-500

India/MH-AFMC-4958/2020/132-500

India/MH-BJMC-1760/2020/132-500

India/MH-BJMC-1123/2020/133-500

SouthKorea/KCDC2030/2020/147-500

SouthKorea/KCDC2066/2020/147-500

SouthKorea/KCDC2069/2020/154-500

SouthKorea/KCDC2070/2020/143-500

SouthKorea/KCDC2073/2020/158-500

SouthKorea/KCDC2074/2020/147-500

SouthKorea/KCDC2095/2020/149-500

SouthKorea/KCDC2101/2020/143-500

England/LIVE-A65D0/2020/131-500

Brazil/PE-IAM215/2020/155-500

Brazil/PE-IAM67/2020/156-500

USA/CT-UW-3422/2020/115-500

USA/VA-DCLS-0683/2020/131-500

Brazil/PE-IAM18/2020/146-500

USA/OR-UW-13394/2020/127-500

USA/UN-UW-13432/2020/125-500

USA/UN-UW-13444/2020/131-500

USA/WA-UW-10161/2020/113-500

USA/WA-UW-10212/2020/131-500

USA/WA-UW-10381/2020/131-500

USA/WA-UW-11089/2020/113-500

USA/WA-UW-11142/2020/113-500

USA/WA-UW-11263/2020/131-500

USA/WA-UW-11300/2020/131-500

USA/WA-UW-11399/2020/131-500

USA/WA-UW-11455/2020/113-500

USA/WA-UW-11538/2020/131-500

USA/WA-UW-11829/2020/113-500

USA/WA-UW-12027/2020/131-500

USA/WA-UW-12149/2020/131-500

USA/WA-UW-12264/2020/117-500

USA/WA-UW-12396/2020/113-500

USA/WA-UW-12596/2020/131-500

USA/WA-UW-12612/2020/113-500

USA/WA-UW-12706/2020/113-500

USA/WA-UW-13045/2020/113-500

USA/WA-UW-13227/2020/113-500

USA/WA-UW-13232/2020/131-500

USA/WA-UW-13233/2020/131-500

USA/WA-UW-13242/2020/113-500

USA/WA-UW-13262/2020/131-500

USA/WA-UW-13264/2020/113-500

USA/WA-UW-13268/2020/113-500

USA/WA-UW-13274/2020/113-500

USA/WA-UW-13275/2020/113-500

USA/WA-UW-13277/2020/113-500

USA/WA-UW-13288/2020/113-500

USA/WA-UW-13291/2020/113-500

USA/WA-UW-13293/2020/113-500

USA/WA-UW-13300/2020/113-500

USA/WA-UW-13310/2020/113-500

USA/WA-UW-13317/2020/113-500

USA/WA-UW-13321/2020/113-500

USA/WA-UW-13330/2020/113-500

USA/WA-UW-13332/2020/113-500

USA/WA-UW-13356/2020/127-500

USA/WA-UW-13364/2020/113-500

USA/WA-UW-13367/2020/113-500

USA/WA-UW-13386/2020/113-500

USA/WA-UW-13395/2020/131-500

USA/WA-UW-13400/2020/113-500

USA/WA-UW-13401/2020/113-500

USA/WA-UW-13402/2020/117-500

USA/WA-UW-7764/2020/113-500

USA/WA-UW-8686/2020/113-500

USA/WA-UW-8803/2020/113-500

USA/WA-UW-8845/2020/113-500

USA/WA-UW-9032/2020/113-500

USA/WA-UW-9291/2020/113-500

USA/WA-UW-9368/2020/113-500

USA/WA-UW-9551/2020/113-500

USA/WA-UW-9755/2020/113-500

USA/WA-UW-9798/2020/113-500

USA/WA-UW-9819/2020/131-500

Malaysia/0121/2020/131-500

Malaysia/3012/2020/131-500

India/UP-AR20/2020/133-500

India/KA-IB38/2020/132-500

India/KA-IB58/2020/132-500

India/WB-IK41/2020/132-500

India/WB-IK66/2020/144-500

Switzerland/ZH-UZH-1000482984/2020/145-500

SouthKorea/KCDC2286/2020/144-500

USA/CA-IGI-0068/2020/133-500

USA/WA-UW-10034/2020/131-500

USA/WA-UW-10105/2020/131-500

USA/WA-UW-10113/2020/131-500

USA/WA-UW-10126/2020/131-500

USA/WA-UW-10133/2020/131-500

SouthKorea/KCDC2454/2020/147-500

SouthKorea/KCDC2527/2020/144-500

SouthKorea/KCDC2530/2020/144-500

SouthKorea/KCDC2539/2020/147-500

SouthKorea/KCDC2559/2020/147-500

SouthKorea/KCDC2572/2020/147-500

SouthKorea/KCDC2574/2020/152-500

SouthKorea/KCDC2579/2020/147-500

SouthKorea/KCDC2586/2020/147-500

SouthKorea/KCDC2590/2020/135-500

SouthKorea/KCDC2592/2020/132-500

SouthKorea/KCDC2595/2020/147-500

SouthKorea/KCDC2597/2020/135-500

SouthKorea/KCDC2598/2020/138-500

SouthKorea/KCDC2629/2020/147-500

SouthKorea/KCDC2650/2020/147-500

SouthKorea/KCDC2651/2020/147-500

Thailand/Nonthaburi_3059/2020/144-500

Thailand/Bangkok_2854/2020/135-500

England/LIVE-A92B8/2020/131-500

SouthKorea/KCDC2732/2020/147-500

USA/VA-DCLS-0799/2020/131-500

Russia/SPE-RII-10152V/2020/134-500

Russia/SPE-RII-11688V/2020/137-500

Russia/SPE-RII-13482V/2020/135-500

Russia/SPE-RII-18966V/2020/134-500

Russia/SPE-RII-19422V/2020/136-500

Russia/SPE-RII-19426V/2020/133-500

Russia/SPE-RII-22068V/2020/135-500

Russia/SPE-RII-22478V/2020/133-500

USA/VA-DCLS-0577/2020/131-500

USA/VA-DCLS-0584/2020/131-500

USA/VA-DCLS-0594/2020/131-500

USA/VA-DCLS-0595/2020/131-500

USA/VA-DCLS-0596/2020/131-500

USA/VA-DCLS-0604/2020/131-500

USA/VA-DCLS-0608/2020/131-500

USA/VA-DCLS-0661/2020/131-500

USA/VA-DCLS-1221/2020/131-500

USA/UT-UPHL-00087/2020/131-500

Australia/QLD987/2020/131-500

USA/MD-MDH-0185/2020/138-500

USA/MD-MDH-0186/2020/131-500

USA/MD-MDH-0191/2020/131-500

Spain/AN-ISCIII-201273/2020/135-500

SouthKorea/IHC17695/2020/147-500

SouthKorea/IHC18561/2020/147-500

England/CAMC-9B1F08/2020/131-500

USA/MD-MDH-0198/2020/132-500

USA/WA-UW-4303/2020/113-500

USA/WA-UW-4331/2020/131-500

USA/WA-UW-4748/2020/115-500

USA/WA-UW-6179/2020/131-500

USA/WA-UW-6204/2020/117-500

USA/ID-UW-6464/2020/131-500

USA/ID-UW-6469/2020/132-500

USA/CT-UW-6684/2020/113-500

USA/WA-UW-6688/2020/127-500

USA/CT-UW-6691/2020/113-500

USA/WA-UW-6706/2020/113-500

USA/CT-UW-6709/2020/113-500

USA/WA-UW-6716/2020/113-500

USA/CT-UW-6758/2020/113-500

USA/WA-UW-6765/2020/113-500

USA/WA-UW-6767/2020/113-500

USA/CT-UW-6780/2020/113-500

USA/WA-UW-6806/2020/113-500

USA/CT-UW-6807/2020/113-500

USA/WA-UW-6817/2020/123-500

USA/WA-UW-6820/2020/113-500

USA/WA-UW-6822/2020/113-500

USA/WA-UW-6971/2020/131-500

USA/WA-UW-6976/2020/131-500

USA/WA-UW-6990/2020/113-500

USA/WA-UW-7006/2020/113-500

USA/WA-UW-7007/2020/113-500

USA/WA-UW-7011/2020/113-500

USA/WA-UW-7012/2020/131-500

USA/WA-UW-7013/2020/131-500

USA/WA-UW-7016/2020/117-500

USA/WA-UW-7022/2020/113-500

USA/WA-UW-7032/2020/113-500

USA/CT-UW-7070/2020/113-500

USA/WA-UW-7106/2020/113-500

USA/WA-UW-7115/2020/113-500

USA/WA-UW-7128/2020/113-500

USA/WA-UW-7158/2020/113-500

USA/WA-UW-7160/2020/113-500

USA/WA-UW-7164/2020/127-500

USA/WA-UW-7188/2020/131-500

USA/WA-UW-7236/2020/113-500

USA/WA-UW-7237/2020/131-500

USA/WA-UW-7242/2020/113-500

USA/CT-UW-7257/2020/113-500

USA/CT-UW-7260/2020/113-500

USA/CT-UW-7265/2020/113-500

USA/CT-UW-7266/2020/131-500

USA/CT-UW-7270/2020/131-500

USA/ID-UW-7283/2020/113-500

USA/CT-UW-7285/2020/113-500

USA/ID-UW-7291/2020/131-500

USA/ID-UW-7306/2020/113-500

USA/ID-UW-7308/2020/113-500

USA/WA-UW-7670/2020/113-500

USA/WA-UW-7671/2020/113-500

USA/WA-UW-8158/2020/113-500

USA/WA-UW-8330/2020/113-500

USA/WA-UW-8418/2020/125-500

USA/WA-UW-8421/2020/113-500

USA/WA-UW-8510/2020/113-500

USA/WA-UW-8511/2020/123-500

USA/WA-UW-8518/2020/127-500

USA/WA-UW-8526/2020/113-500

USA/WA-UW-8550/2020/131-500

USA/WA-UW-8638/2020/121-500

USA/WA-UW-8698/2020/131-500

USA/WA-UW-8800/2020/113-500

USA/WA-UW-8813/2020/113-500

USA/WA-UW-8824/2020/113-500

USA/WA-UW-8831/2020/113-500

USA/WA-UW-8872/2020/113-500

USA/WA-UW-8873/2020/113-500

USA/WA-UW-9014/2020/113-500

USA/WA-UW-9068/2020/113-500

USA/WA-UW-9069/2020/113-500

USA/WA-UW-9190/2020/113-500

USA/WA-UW-9195/2020/113-500

USA/WA-UW-9214/2020/113-500

USA/WA-UW-9227/2020/113-500

USA/WA-UW-9228/2020/113-500

USA/WA-UW-9229/2020/113-500

USA/WA-UW-9230/2020/113-500

USA/WA-UW-9233/2020/113-500

USA/WA-UW-9235/2020/113-500

USA/WA-UW-9244/2020/113-500

USA/WA-UW-9255/2020/113-500

USA/WA-UW-9266/2020/133-500

USA/WA-UW-9301/2020/131-500

USA/WA-UW-9308/2020/113-500

USA/WA-UW-9333/2020/113-500

USA/WA-UW-9338/2020/113-500

USA/WA-UW-9339/2020/113-500

USA/OR-UW-9346/2020/113-500

USA/WA-UW-9361/2020/113-500

USA/WA-UW-9374/2020/113-500

USA/WA-UW-9390/2020/113-500

USA/WA-UW-9407/2020/113-500

USA/WA-UW-9422/2020/113-500

USA/WA-UW-9439/2020/113-500

USA/WA-UW-9460/2020/113-500

USA/WA-UW-9500/2020/131-500

USA/WA-UW-9520/2020/113-500

USA/WA-UW-9532/2020/113-500

USA/WA-UW-9534/2020/113-500

USA/WA-UW-9536/2020/113-500

USA/WA-UW-9568/2020/113-500

USA/WA-UW-9569/2020/113-500

USA/WA-UW-9578/2020/113-500

USA/WA-UW-9579/2020/113-500

USA/WA-UW-9580/2020/113-500

USA/WA-UW-9600/2020/113-500

USA/WA-UW-10110/2020/113-500

USA/WA-UW-10159/2020/113-500

USA/WA-UW-10163/2020/113-500

USA/WA-UW-10185/2020/113-500

USA/WA-UW-10190/2020/113-500

USA/WA-UW-10191/2020/131-500

USA/WA-UW-10197/2020/131-500

USA/WA-UW-10199/2020/113-500

USA/WA-UW-10206/2020/113-500

USA/WA-UW-10213/2020/131-500

USA/WA-UW-10216/2020/131-500

USA/WA-UW-10217/2020/113-500

USA/WA-UW-10230/2020/113-500

USA/WA-UW-10247/2020/131-500

USA/WA-UW-10250/2020/113-500

USA/WA-UW-10272/2020/131-500

USA/WA-UW-10277/2020/113-500

USA/WA-UW-10283/2020/113-500

USA/WA-UW-10284/2020/113-500

USA/WA-UW-10286/2020/131-500

USA/WA-UW-10298/2020/131-500

USA/WA-UW-10305/2020/113-500

USA/WA-UW-10306/2020/113-500

USA/WA-UW-10317/2020/131-500

USA/WA-UW-10339/2020/131-500

USA/WA-UW-10365/2020/113-500

USA/WA-UW-10380/2020/113-500

USA/WA-UW-10392/2020/131-500

USA/WA-UW-10394/2020/113-500

USA/WA-UW-10396/2020/113-500

USA/WA-UW-10402/2020/131-500

USA/WA-UW-10403/2020/131-500

USA/WA-UW-10408/2020/131-500

USA/OR-UW-10424/2020/131-500

USA/WA-UW-10435/2020/131-500

USA/WA-UW-10438/2020/113-500

USA/WA-UW-10439/2020/131-500

USA/WA-UW-10442/2020/131-500

USA/WA-UW-10447/2020/131-500

USA/WA-UW-10449/2020/113-500

USA/WA-UW-10453/2020/131-500

USA/WA-UW-10455/2020/113-500

USA/WA-UW-10459/2020/113-500

USA/WA-UW-10464/2020/131-500

USA/WA-UW-10465/2020/113-500

USA/WA-UW-10471/2020/113-500

USA/WA-UW-10472/2020/113-500

USA/WA-UW-10473/2020/131-500

USA/WA-UW-10479/2020/131-500

USA/WA-UW-10485/2020/131-500

USA/WA-UW-10496/2020/113-500

USA/WA-UW-10501/2020/131-500

USA/WA-UW-10508/2020/113-500

USA/WA-UW-10510/2020/113-500

USA/WA-UW-10511/2020/113-500

USA/WA-UW-10512/2020/131-500

USA/WA-UW-10521/2020/113-500

USA/WA-UW-10530/2020/113-500

USA/WA-UW-10533/2020/131-500

USA/WA-UW-10552/2020/113-500

USA/WA-UW-10588/2020/131-500

USA/WA-UW-10599/2020/125-500

USA/WA-UW-10609/2020/113-500

USA/WA-UW-10612/2020/113-500

USA/WA-UW-10621/2020/113-500

USA/WA-UW-10648/2020/131-500

USA/WA-UW-10652/2020/131-500

USA/WA-UW-10654/2020/113-500

USA/WA-UW-10655/2020/123-500

USA/WA-UW-10658/2020/113-500

USA/WA-UW-10663/2020/125-500

USA/WA-UW-10681/2020/131-500

USA/WA-UW-10685/2020/113-500

USA/WA-UW-10687/2020/131-500

USA/WA-UW-10688/2020/113-500

USA/WA-UW-10691/2020/131-500

USA/WA-UW-10696/2020/129-500

USA/WA-UW-10697/2020/131-500

USA/WA-UW-10698/2020/131-500

USA/WA-UW-11059/2020/113-500

USA/WA-UW-11090/2020/131-500

USA/WA-UW-11098/2020/131-500

USA/WA-UW-11289/2020/114-500

USA/WA-UW-11290/2020/131-500

USA/WA-UW-11341/2020/115-500

USA/WA-UW-11394/2020/131-500

USA/WA-UW-11401/2020/114-500

USA/WA-UW-11472/2020/131-500

USA/WA-UW-11480/2020/131-500

USA/WA-UW-11488/2020/117-500

USA/WA-UW-11513/2020/114-500

USA/WA-UW-11519/2020/131-500

USA/WA-UW-11526/2020/116-500

USA/WA-UW-11537/2020/131-500

USA/WA-UW-11589/2020/131-500

USA/WA-UW-11602/2020/131-500

USA/WA-UW-11611/2020/131-500

USA/WA-UW-11728/2020/115-500

USA/WA-UW-11763/2020/131-500

USA/WA-UW-12283/2020/113-500

USA/WA-UW-12333/2020/131-500

USA/WA-UW-12348/2020/113-500

USA/WA-UW-12356/2020/131-500

USA/WA-UW-12360/2020/115-500

USA/WA-UW-12361/2020/113-500

USA/WA-UW-12362/2020/131-500

USA/WA-UW-12364/2020/131-500

USA/WA-UW-12367/2020/131-500

USA/WA-UW-12377/2020/131-500

USA/WA-UW-12378/2020/131-500

USA/WA-UW-12384/2020/131-500

USA/WA-UW-12389/2020/113-500

USA/WA-UW-12390/2020/113-500

USA/WA-UW-12391/2020/113-500

USA/WA-UW-12392/2020/131-500

USA/WA-UW-12395/2020/113-500

USA/WA-UW-12398/2020/129-500

USA/WA-UW-12400/2020/113-500

USA/WA-UW-12401/2020/131-500

USA/WA-UW-12437/2020/131-500

USA/WA-UW-12440/2020/131-500

USA/WA-UW-12478/2020/113-500

USA/WA-UW-12488/2020/113-500

USA/WA-UW-12516/2020/113-500

USA/WA-UW-12521/2020/115-500

USA/WA-UW-12531/2020/113-500

USA/WA-UW-12539/2020/131-500

USA/WA-UW-12543/2020/113-500

USA/WA-UW-12548/2020/113-500

USA/WA-UW-22535/2020/131-500

USA/WA-UW-24798/2020/131-500

USA/WA-UW-22270/2020/131-500

USA/WA-UW-22938/2020/113-500

USA/WA-UW-26847/2020/131-500

USA/WA-UW-26853/2020/131-500

USA/WA-UW-26854/2020/113-500

USA/WA-UW-26863/2020/113-500

USA/WA-UW-26866/2020/131-500

USA/WA-UW-22048/2020/115-500

USA/WA-UW-22090/2020/113-500

USA/WA-UW-22117/2020/131-500

USA/WA-UW-22230/2020/113-500

USA/WA-UW-22057/2020/131-500

USA/WA-UW-22060/2020/131-500

USA/WA-UW-22103/2020/113-500

USA/WA-UW-22137/2020/113-500

USA/WA-UW-22155/2020/131-500

USA/WA-UW-22162/2020/113-500

USA/WA-UW-22172/2020/131-500

USA/WA-UW-23194/2020/113-500

USA/WA-UW-22847/2020/131-500

USA/WA-UW-22256/2020/113-500

USA/WA-UW-22817/2020/113-500

USA/WA-UW-24887/2020/131-500

USA/WA-UW-27035/2020/113-500

USA/WA-UW-24946/2020/113-500

USA/WA-UW-22855/2020/113-500

USA/WA-UW-26495/2020/113-500

USA/WA-UW-22460/2020/131-500

USA/WA-UW-26865/2020/120-500

USA/WA-UW-24802/2020/121-500

USA/WA-UW-22353/2020/119-500

USA/WA-UW-24920/2020/113-500

USA/OR-UW-24378/2020/113-500

USA/WA-UW-22056/2020/113-500

USA/WA-UW-22190/2020/113-500

USA/WA-UW-22205/2020/113-500

USA/WA-UW-22216/2020/113-500

USA/WA-UW-22236/2020/131-500

USA/WA-UW-22271/2020/113-500

USA/WA-UW-22415/2020/131-500

USA/WA-UW-22521/2020/131-500

USA/WA-UW-22809/2020/131-500

USA/WA-UW-23335/2020/131-500

USA/WA-UW-23564/2020/131-500

USA/WA-UW-23691/2020/113-500

USA/WA-UW-23752/2020/113-500

USA/WA-UW-23782/2020/131-500

USA/WA-UW-24322/2020/131-500

USA/WA-UW-24958/2020/131-500

USA/WA-UW-26856/2020/131-500

USA/WA-UW-22346/2020/131-500

USA/WA-UW-22348/2020/113-500

USA/WA-UW-22451/2020/131-500

USA/WA-UW-24890/2020/131-500

USA/WA-UW-22731/2020/113-500

USA/WA-UW-22852/2020/131-500

USA/WA-UW-24891/2020/131-500

USA/OR-UW-22276/2020/123-500

USA/OR-UW-22416/2020/113-500

USA/OR-UW-22446/2020/131-500

USA/OR-UW-22452/2020/113-500

USA/OR-UW-22482/2020/113-500

USA/OR-UW-22871/2020/113-500

USA/OR-UW-22918/2020/113-500

USA/WA-UW-22051/2020/113-500

USA/WA-UW-22061/2020/131-500

USA/WA-UW-22077/2020/113-500

USA/WA-UW-22080/2020/113-500

USA/WA-UW-22094/2020/113-500

USA/WA-UW-22096/2020/113-500

USA/WA-UW-22112/2020/113-500

USA/WA-UW-22124/2020/131-500

USA/WA-UW-22144/2020/113-500

USA/WA-UW-22148/2020/113-500

USA/WA-UW-22149/2020/113-500

USA/WA-UW-22151/2020/113-500

USA/WA-UW-22157/2020/131-500

USA/WA-UW-22173/2020/113-500

USA/WA-UW-22175/2020/131-500

USA/WA-UW-22177/2020/131-500

USA/WA-UW-22180/2020/113-500

USA/WA-UW-22201/2020/131-500

USA/WA-UW-22228/2020/113-500

USA/WA-UW-22253/2020/113-500

USA/WA-UW-22258/2020/113-500

USA/WA-UW-22273/2020/113-500

USA/WA-UW-22275/2020/113-500

USA/WA-UW-22286/2020/131-500

USA/WA-UW-22293/2020/113-500

USA/WA-UW-22312/2020/131-500

USA/WA-UW-22313/2020/131-500

USA/WA-UW-22316/2020/131-500

USA/WA-UW-22327/2020/113-500

USA/WA-UW-22328/2020/113-500

USA/WA-UW-22330/2020/131-500

USA/WA-UW-22331/2020/131-500

USA/WA-UW-22339/2020/113-500

USA/WA-UW-22343/2020/131-500

USA/WA-UW-22358/2020/131-500

USA/WA-UW-22360/2020/113-500

USA/WA-UW-22363/2020/131-500

USA/WA-UW-22365/2020/113-500

USA/WA-UW-22370/2020/113-500

USA/WA-UW-22376/2020/131-500

USA/WA-UW-22382/2020/131-500

USA/WA-UW-22389/2020/113-500

USA/WA-UW-22398/2020/131-500

USA/WA-UW-22399/2020/131-500

USA/WA-UW-22405/2020/131-500

USA/WA-UW-22407/2020/131-500

USA/WA-UW-22409/2020/131-500

USA/WA-UW-22412/2020/131-500

USA/WA-UW-22418/2020/131-500

USA/WA-UW-22422/2020/113-500

USA/WA-UW-22426/2020/113-500

USA/WA-UW-22429/2020/113-500

USA/WA-UW-22431/2020/131-500

USA/WA-UW-22432/2020/131-500

USA/WA-UW-22436/2020/131-500

USA/WA-UW-22445/2020/131-500

USA/WA-UW-22447/2020/131-500

USA/WA-UW-22449/2020/131-500

USA/WA-UW-22454/2020/131-500

USA/WA-UW-22458/2020/131-500

USA/WA-UW-22476/2020/131-500

USA/WA-UW-22491/2020/131-500

USA/WA-UW-22493/2020/113-500

USA/WA-UW-22495/2020/131-500

USA/WA-UW-22505/2020/129-500

USA/WA-UW-22514/2020/131-500

USA/WA-UW-22517/2020/131-500

USA/WA-UW-22720/2020/131-500

USA/WA-UW-22735/2020/131-500

USA/WA-UW-22746/2020/131-500

USA/WA-UW-22747/2020/131-500

USA/WA-UW-22748/2020/113-500

USA/WA-UW-22751/2020/131-500

USA/WA-UW-22752/2020/131-500

USA/WA-UW-22753/2020/131-500

USA/WA-UW-22756/2020/113-500

USA/WA-UW-22757/2020/121-500

USA/WA-UW-22758/2020/113-500

USA/WA-UW-22762/2020/113-500

USA/WA-UW-22769/2020/115-500

USA/WA-UW-22774/2020/113-500

USA/WA-UW-22782/2020/113-500

USA/WA-UW-22784/2020/113-500

USA/WA-UW-22791/2020/113-500

USA/WA-UW-22804/2020/131-500

USA/WA-UW-22827/2020/131-500

USA/WA-UW-22835/2020/113-500

USA/WA-UW-22839/2020/121-500

USA/WA-UW-22848/2020/113-500

USA/WA-UW-22851/2020/113-500

USA/WA-UW-22854/2020/113-500

USA/WA-UW-22859/2020/113-500

USA/WA-UW-22867/2020/115-500

USA/WA-UW-22899/2020/113-500

USA/WA-UW-22900/2020/113-500

USA/WA-UW-22903/2020/115-500

USA/WA-UW-22920/2020/113-500

USA/WA-UW-22922/2020/113-500

USA/WA-UW-22951/2020/115-500

USA/WA-UW-22952/2020/113-500

USA/WA-UW-22963/2020/113-500

USA/WA-UW-22972/2020/131-500

USA/WA-UW-22978/2020/113-500

USA/WA-UW-22993/2020/113-500

USA/WA-UW-22994/2020/125-500

USA/WA-UW-22998/2020/113-500

USA/WA-UW-23004/2020/131-500

USA/WA-UW-23124/2020/131-500

USA/WA-UW-23130/2020/113-500

USA/WA-UW-23152/2020/113-500

USA/WA-UW-23184/2020/131-500

USA/WA-UW-23193/2020/113-500

USA/WA-UW-23196/2020/131-500

USA/WA-UW-23200/2020/131-500

USA/WA-UW-23206/2020/113-500

USA/WA-UW-23213/2020/131-500

USA/WA-UW-23483/2020/131-500

USA/WA-UW-23756/2020/131-500

USA/WA-UW-24373/2020/131-500

USA/WA-UW-24775/2020/113-500

USA/WA-UW-24787/2020/131-500

USA/WA-UW-24839/2020/113-500

USA/WA-UW-24901/2020/113-500

USA/WA-UW-24925/2020/113-500

USA/WA-UW-24930/2020/131-500

USA/WA-UW-24935/2020/131-500

USA/WA-UW-24937/2020/113-500

USA/WA-UW-24941/2020/131-500

USA/WA-UW-26858/2020/113-500

USA/WA-UW-26860/2020/113-500

USA/WA-UW-26861/2020/131-500

USA/WA-UW-26862/2020/111-500

USA/WA-UW-26867/2020/113-500

USA/WA-UW-26868/2020/113-500

USA/WA-UW-27032/2020/125-500

USA/WA-UW-27033/2020/113-500

USA/WA-UW-27034/2020/131-500

USA/WA-UW-27038/2020/113-500

USA/WA-UW-27042/2020/113-500

USA/WA-UW-27498/2020/113-500

USA/WA-UW-27499/2020/113-500

USA/WA-UW-27500/2020/113-500

USA/WA-UW-27501/2020/131-500

USA/WA-UW-12874/2020/113-500

USA/WA-UW-19700/2020/113-500

USA/WA-UW-19395/2020/113-500

USA/WA-UW-21754/2020/113-500

USA/WA-UW-19249/2020/113-500

USA/WA-UW-12831/2020/113-500

USA/WA-UW-21673/2020/113-500

USA/WA-UW-21969/2020/131-500

USA/WA-UW-19536/2020/131-500

USA/WA-UW-21712/2020/113-500

USA/WA-UW-21995/2020/131-500

USA/WA-UW-12632/2020/113-500

USA/WA-UW-12657/2020/113-500

USA/WA-UW-21747/2020/131-500

USA/WA-UW-21764/2020/131-500

USA/WA-UW-22027/2020/113-500

USA/WA-UW-22028/2020/131-500

USA/WA-UW-13090/2020/120-500

USA/WA-UW-12601/2020/113-500

USA/WA-UW-13016/2020/131-500

USA/WA-UW-22017/2020/131-500

USA/WA-UW-21757/2020/131-500

USA/WA-UW-21759/2020/131-500

USA/WA-UW-12796/2020/113-500

USA/WA-UW-21409/2020/113-500

USA/WA-UW-13047/2020/113-500

USA/WA-UW-21721/2020/131-500

USA/ID-UW-21755/2020/113-500

USA/OR-UW-12646/2020/131-500

USA/WA-UW-13031/2020/113-500

USA/WA-UW-13081/2020/113-500

USA/WA-UW-19260/2020/113-500

USA/WA-UW-19262/2020/131-500

USA/WA-UW-19376/2020/113-500

USA/WA-UW-21726/2020/113-500

USA/WA-UW-21789/2020/131-500

USA/WA-UW-22009/2020/131-500

USA/WA-UW-12772/2020/115-500

USA/WA-UW-12641/2020/113-500

USA/WA-UW-21728/2020/114-500

USA/OR-UW-12645/2020/131-500

USA/WA-UW-12557/2020/113-500

USA/WA-UW-12570/2020/131-500

USA/WA-UW-12575/2020/131-500

USA/WA-UW-12576/2020/113-500

USA/WA-UW-12593/2020/113-500

USA/WA-UW-12600/2020/131-500

USA/WA-UW-12602/2020/113-500

USA/WA-UW-12604/2020/113-500

USA/WA-UW-12611/2020/131-500

USA/WA-UW-12613/2020/131-500

USA/WA-UW-12624/2020/131-500

USA/WA-UW-12625/2020/131-500

USA/WA-UW-12626/2020/113-500

USA/WA-UW-12634/2020/113-500

USA/WA-UW-12637/2020/131-500

USA/WA-UW-12685/2020/113-500

USA/WA-UW-12709/2020/113-500

USA/WA-UW-12716/2020/131-500

USA/WA-UW-12728/2020/113-500

USA/WA-UW-12732/2020/113-500

USA/WA-UW-12733/2020/113-500

USA/WA-UW-12760/2020/113-500

USA/WA-UW-12761/2020/113-500

USA/WA-UW-12801/2020/113-500

USA/WA-UW-12822/2020/113-500

USA/WA-UW-12830/2020/113-500

USA/WA-UW-12832/2020/113-500

USA/WA-UW-12839/2020/113-500

USA/WA-UW-12846/2020/113-500

USA/WA-UW-12858/2020/113-500

USA/WA-UW-12863/2020/113-500

USA/WA-UW-12875/2020/113-500

USA/WA-UW-12878/2020/131-500

USA/WA-UW-12879/2020/113-500

USA/WA-UW-12883/2020/113-500

USA/WA-UW-12886/2020/113-500

USA/WA-UW-12898/2020/113-500

USA/WA-UW-12907/2020/113-500

USA/WA-UW-12908/2020/113-500

USA/WA-UW-12909/2020/113-500

USA/WA-UW-12926/2020/113-500

USA/WA-UW-12930/2020/123-500

USA/WA-UW-12934/2020/113-500

USA/WA-UW-12936/2020/113-500

USA/WA-UW-12948/2020/113-500

USA/WA-UW-12957/2020/131-500

USA/WA-UW-12962/2020/113-500

USA/WA-UW-12978/2020/113-500

USA/WA-UW-13015/2020/113-500

USA/WA-UW-13027/2020/131-500

USA/WA-UW-13029/2020/113-500

USA/WA-UW-13068/2020/131-500

USA/WA-UW-13071/2020/113-500

USA/WA-UW-13082/2020/131-500

USA/WA-UW-13083/2020/123-500

USA/WA-UW-13084/2020/113-500

USA/WA-UW-13085/2020/113-500

USA/WA-UW-13088/2020/113-500

USA/WA-UW-13201/2020/113-500

USA/WA-UW-13219/2020/113-500

USA/WA-UW-1859/2020/115-500

USA/WA-UW-19384/2020/131-500

USA/WA-UW-19418/2020/131-500

USA/WA-UW-19676/2020/113-500

USA/WA-UW-21707/2020/131-500

USA/WA-UW-21710/2020/113-500

USA/WA-UW-21720/2020/131-500

USA/WA-UW-21727/2020/131-500

USA/WA-UW-21732/2020/131-500

USA/WA-UW-21734/2020/113-500

USA/WA-UW-21735/2020/113-500

USA/WA-UW-21736/2020/131-500

USA/WA-UW-21742/2020/131-500

USA/WA-UW-21746/2020/131-500

USA/WA-UW-21752/2020/131-500

USA/WA-UW-21753/2020/131-500

USA/WA-UW-21756/2020/131-500

USA/WA-UW-21771/2020/131-500

USA/WA-UW-21784/2020/131-500

USA/WA-UW-21786/2020/131-500

USA/WA-UW-21960/2020/131-500

USA/WA-UW-21985/2020/131-500

USA/WA-UW-21986/2020/131-500

USA/WA-UW-21987/2020/131-500

USA/WA-UW-21998/2020/113-500

USA/WA-UW-22003/2020/131-500

USA/WA-UW-22016/2020/113-500

USA/WA-UW-22038/2020/113-500

USA/WA-UW-22039/2020/131-500

USA/VA-DCLS-1314/2020/131-500

England/ALDP-9BCBCD/2020/133-500

USA/UT-UPHL-01361/2020/131-500

USA/UT-UPHL-01644/2020/131-500

Indonesia/JT-UGM-47906/2020/131-500

USA/UN-UW-10411/2020/131-500

USA/UN-UW-12782/2020/113-500

USA/WA-UW-12787/2020/113-500

USA/UN-UW-7167/2020/113-500

USA/UN-UW-7187/2020/113-500

USA/UN-UW-7196/2020/113-500

USA/UN-UW-7204/2020/113-500

USA/UN-UW-7214/2020/113-500

USA/UN-UW-7220/2020/113-500

USA/UN-UW-7227/2020/113-500

USA/UN-UW-7228/2020/113-500

USA/UN-UW-7230/2020/113-500

USA/UN-UW-7231/2020/113-500

USA/UN-UW-7424/2020/113-500

USA/UN-UW-7428/2020/113-500

USA/UN-UW-7434/2020/113-500

USA/UN-UW-7435/2020/131-500

USA/UN-UW-7455/2020/113-500

USA/UN-UW-7473/2020/131-500

England/ALDP-9D98F0/2020/148-500

Spain/AN-ISCIII-2013382/2020/131-500

USA/VA-DCLS-1525/2020/131-500

USA/VA-DCLS-1545/2020/131-500

USA/VA-DCLS-1481/2020/131-500

USA/VA-DCLS-1490/2020/131-500

USA/VA-DCLS-1492/2020/131-500

USA/MD-MDH-0224/2020/131-500

USA/UT-UPHL-201006663/2020/131-500

USA/UT-UPHL-201004959/2020/131-500

USA/UT-UPHL-201002768/2020/131-500

USA/WA-UW-24961/2020/113-500

USA/VA-DCLS-1561/2020/131-500

USA/VA-DCLS-1985/2020/131-500

USA/VA-DCLS-1991/2020/131-500

USA/VA-DCLS-0311/2020/131-500

USA/VA-DCLS-1818/2020/131-500
